# PSP-GNM: Predicting Protein Stability Changes upon Point Mutations with a Gaussian Network Model

**DOI:** 10.3390/ijms231810711

**Published:** 2022-09-14

**Authors:** Sambit Kumar Mishra

**Affiliations:** 1 Cancer Genomics Research Laboratory, Leidos Biomedical Research, Inc., Rockville, MD 20850, USA; sambit.mishra@nih.gov; 2 Division of Cancer Epidemiology and Genetics, National Cancer Institute, Bethesda, MD 20892, USA

**Keywords:** Gaussian network models, missense mutations, protein stability, Gibbs free energy change, Miyazawa–Jernigan potential

## Abstract

Understanding the effects of missense mutations on protein stability is a widely acknowledged significant biological problem. Genomic missense mutations may alter one or more amino acids, leading to increased or decreased stability of the encoded proteins. In this study, we describe a novel approach—Protein Stability Prediction with a Gaussian Network Model (PSP-GNM)—to measure the unfolding Gibbs free energy change (ΔΔG) and evaluate the effects of single amino acid substitutions on protein stability. Specifically, PSP-GNM employs a coarse-grained Gaussian Network Model (GNM) that has interactions between amino acids weighted by the Miyazawa–Jernigan statistical potential. We used PSP-GNM to simulate partial unfolding of the wildtype and mutant protein structures, and then used the difference in the energies and entropies of the unfolded wildtype and mutant proteins to calculate ΔΔG. The extent of the agreement between the ΔΔG calculated by PSP-GNM and the experimental ΔΔG was evaluated on three benchmark datasets: 350 forward mutations (S350 dataset), 669 forward and reverse mutations (S669 dataset) and 611 forward and reverse mutations (S611 dataset). We observed a Pearson correlation coefficient as high as 0.61, which is comparable to many of the existing state-of-the-art methods. The agreement with experimental ΔΔG further increased when we considered only those measurements made close to 25 °C and neutral pH, suggesting dependence on experimental conditions. We also assessed for the antisymmetry (ΔΔG*_reverse_* = −ΔΔG*_forward_*) between the forward and reverse mutations on the Ssym+ dataset, which has 352 forward and reverse mutations. While most available methods do not display significant antisymmetry, PSP-GNM demonstrated near-perfect antisymmetry, with a Pearson correlation of −0.97. PSP-GNM is written in Python and can be downloaded as a stand-alone code.

## 1. Introduction

Genomic mutations are often random and can either be synonymous mutations that do not alter the amino acid sequence of an encoded protein or non-synonymous mutations that alter the amino acid sequence. Non-synonymous point mutations can lead to structural changes, affecting the folding energy landscape and thermodynamic stability of proteins [1,2,3,4]. They may sometimes have a neutral effect on the folding energy landscape of proteins; that is, there may be little difference in the thermodynamic stability of the mutant and wildtype proteins. However, mutations often tend to change the folding energy landscape, rendering the mutant proteins either more or less stable compared to the wildtype [5,6]. Understanding the impact of non-synonymous point mutations on the thermodynamic stability of proteins is regarded as a key biological problem. For instance, many neurodegenerative diseases and genetic disorders have been linked to the incorrect folding of polypeptides that is caused by mutations in genes [7]. It is therefore necessary to understand how non-synonymous mutations impact the thermodynamic stability of proteins to better associate their roles in different diseases.

Mutagenesis experiments that assess the change in the thermodynamic stability of proteins measure the energies of the folded and unfolded states of the wildtype and mutant proteins, providing an accurate picture of stability change upon mutations. However, such experiments are time-consuming and expensive; thus, there is a need for alternative computational methods that accurately predict the effects of mutations on protein stability. Databases such as ProTherm [8], ThermoMutDB [9] and FireProtDB [10] have mined such experimental data from the literature and have recorded details of changes in free energies (ΔΔG) upon mutations. Experimental ΔΔG is the difference in the Gibbs free energies (ΔG) of the mutant and wildtype proteins. Specifically, $∆∆G=∆G_{mut}-∆G_{wt}$, where $∆G_{mut}$ is the energy of the unfolded state minus the energy of the folded state of the mutant protein ($∆G_{mut}^{unfolded}-∆G_{mut}^{folded}$), while $∆G_{wt}$ is the energy of the unfolded state minus the energy of the folded state of the wildtype protein ($∆G_{wt}^{unfolded}-∆G_{wt}^{folded}$) [11]. A positive ΔΔG indicates greater thermodynamic stability of the mutant protein, while a negative ΔΔG indicates reduced stability. It is to be noted that forward mutants (wildtype residue X → mutant residue Y) are antisymmetric to their corresponding reverse mutants (mutant residue Y → wildtype residue X) in terms of their folding free energies [12,13]. That is, ΔΔG*_forward_* = −ΔΔG*_reverse_*, which is a key outcome from mutagenesis experiments and is often used to evaluate predictions for ΔΔG from computational methods.

Numerous computational methods have been developed to predict the ΔΔG between a wildtype protein and its non-synonymous mutant form. The review articles by Marabotti et al. [14] and Sanavia et al. [15] provide a comprehensive overview of the computational methods developed towards this effort. Existing methods may be broadly classified into two categories: unsupervised and supervised methods. Unsupervised methods are untrained methods and do not rely on machine learning. They can include empirical methods that model the energy of a protein through an equation that includes several parameters, with each parameter carrying a different weight. For example, the FoldX empirical method [16] uses both the bonded and non-bonded energy terms to model a protein’s energy, and then calculates the difference in the energies of the mutant and wildtype proteins to estimate the ΔΔG. SDM [17] uses environment-specific amino acid substitution tables and residue packing densities to calculate the ΔΔG. DDGun is another unsupervised method that uses sequence and structural properties of proteins in linear weighted combinations to estimate the ΔΔG [18]. Specifically, it is one of the few methods that demonstrate the antisymmetric relationship between forward and reverse mutants. Supervised methods employ machine learning and are trained on a large dataset (training data) including features (e.g., amino acid physicochemical properties, protein geometric properties, etc.) of the wildtype and mutant forms of different proteins and their associated experimental ΔΔG. Such methods are then typically evaluated on an independent dataset of wildtype and mutant proteins with known experimental ΔΔG.

Numerous supervised computational methods to predict stability changes in proteins upon mutations have been developed in the past decade. These methods often employ machine learning models trained on diverse benchmark datasets obtained from the ProTherm database. I-mutant [19,20] employs support vector machines (SVMs) [21] trained on a subset of data taken from ProTherm and uses a set of sequence and spatial features, including solvent accessibility, to predict the ΔΔG. It also gives the user an option to make predictions using just the protein sequence in case the protein structure is not available. mCSM [22] represents the environment of the mutation site as a distance-based graphical map and combines it with pharmacophore counts to predict the effect of mutations using a trained linear regression model. MAESTRO [23] uses statistical scoring functions and other sequence and structural features, such as the accessible surface area, hydrophobicity and secondary structure of the mutation site, to train artificial neural networks, SVMs and multiple linear regression models, making consensus ensemble-based predictions. Dynamut [24] follows a meta-prediction approach by using predictions from other methods, which use protein structure and dynamics information to estimate ΔΔG, to arrive at a consensus prediction for ΔΔG. Dynamut2 [25] predicts ΔΔG using a Random Forest algorithm trained on protein dynamics features calculated from normal mode analysis and graph-based signatures.

It has generally been observed that supervised computational methods outperform unsupervised methods in predicting ΔΔG [25]. However, one of the key shortcomings of the supervised methods is overfitting the training data, which largely comprises destabilizing mutations. Consequently, only a handful of these methods satisfy the antisymmetric property between forward and reverse mutations. Some of the more recently developed supervised approaches, such as PremPS [26], ACDC-NN [27] and Thermonet [28] have addressed this issue by training on both forward and reverse mutation data. Moreover, supervised methods use an ensemble of features that are selected purely based on the algorithms’ performance in cross-validation, not based on any theoretical model that simulates the mutational perturbation behavior. It is always a challenge to associate such features with mutational perturbation, understand how a mutation affects each of these features and explain the biological significance and role of each feature in the context of protein thermodynamic stability.

In this study, we present a theoretical and unsupervised method—PSP-GNM, which simulates protein unfolding behavior using the Gaussian Network Model (GNM) [29] and calculates ΔΔG for a pair of wildtype and mutant proteins. GNM is an elastic network model that models a protein using a coarse-grained representation. Each amino acid in GNM is represented by its alpha carbon, and the interacting amino acid pairs are connected using hypothetical Hookean springs [29]. Previously, elastic network models have been shown to capture the near-native dynamics of proteins [30] and have been used to efficiently identify functional sites in proteins [31,32]. GNM has been previously employed to study changes in protein dynamic communities upon mutations and has been shown to possess the ability to identify stabilizing and destabilizing mutations in proteins [33]. In addition, GNM has also been previously utilized to study protein unfolding behavior and identify the order of contacts broken during protein unfolding [34,35]. PSP-GNM utilizes the knowledge of amino acid contacts that are broken at the mutation site during theoretical protein unfolding to estimate the energy change. It is based on protein dynamics obtained using a coarse-grained representation of the protein and provides useful insights into the importance of the local dynamics of the mutation site and its interactions with neighbors, helping us to understand protein stability changes upon mutation. We evaluated the calculated ΔΔG from PSP-GNM on multiple benchmark datasets obtained from the ProTherm database and compared the predictions from PSP-GNM with other state-of-the-art methods. Despite being an unsupervised approach, PSP-GNM shows comparable performance to many supervised approaches.

## 2. Results

PSP-GNM utilizes the coarse-grained Gaussian Network Model (GNM) to simulate partial protein unfolding. Unlike the conventional GNM, which has interacting residues connected with springs that have a uniform force constant of one, PSP-GNM weights the interacting residues using their interaction energies obtained from the Miyazawa–Jernigan potential [36]. We chose the Miyazawa–Jernigan potential for two reasons. First, a pilot analysis (Table S1) suggested superior performance of the Miyazawa–Jernigan potential compared to two other potentials: the Bastolla potential [37] and the Betancourt–Thirumalai potential [38]. Second, the Miyazawa–Jernigan potential is a very popular choice with a wide variety of applications and has been shown to outperform other potentials in protein–protein docking [39] and protein folding [38].

To simulate partial protein unfolding, the residue–residue contacts with the highest mean-squared fluctuations (MSFs) in their distance are first identified, both in the wildtype and mutant proteins. In this context, MSF is the average change in internal distance for a given pair of residues. These contacts are then removed from the amino acid contact matrices of the wildtype and mutant. The new contact matrix is then used for MSF calculations in the next iteration, and the same procedure of identifying and removing the contacts with the highest MSF is followed. We refer to this method of sequentially identifying high-MSF contacts and removing them as protein unfolding. Previous studies have successfully used this approach to study protein unfolding behavior [34,35]. We consider a protein to be partially unfolded when 50% of all contacts observed in the starting structure are broken. Contacts broken with the residue at the mutation position are identified and ranked in the order they were broken. Broken contacts that share the same rank for a pair of mutant and wildtype proteins are then considered for calculations of ΔΔG.

**Figure 1:**
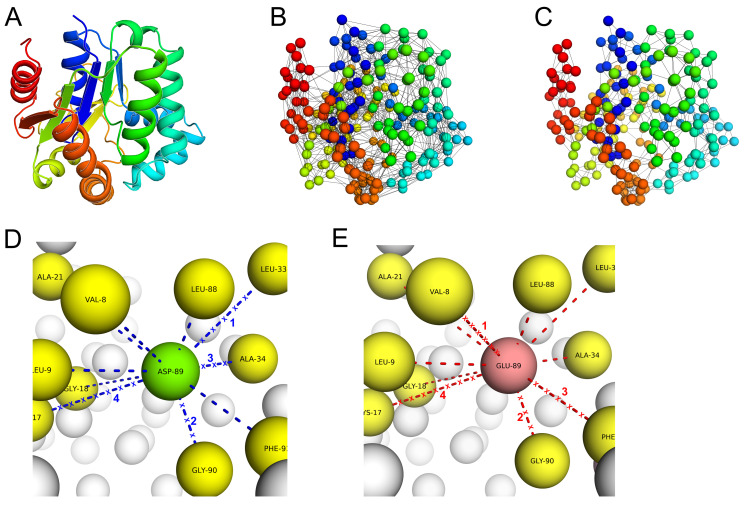
**An overview of the PSP-GNM approach.** (**A**) The starting structure is an all-atom PDB structure. (**B**) The PDB structure is coarse-grained into a bead-and-spring representation. Each amino acid is represented as a bead, and the interactions with other amino acids within a cutoff distance are represented as springs (lines). The interactions between pairs of residues are weighted by their corresponding Miyazawa–Jernigan potentials. Both wildtype and mutant proteins are coarse-grained. (**C**) Both mutant and wildtype forms are simulated for unfolding. Residue–residue contacts that have the highest MSF in their distance are iteratively identified and removed. The iteration is performed until 50% of contacts in the starting structure are broken. (**D**) The native state contacts (blue dashed lines) for the residue at the mutation position (ASP-89) and the contacts broken (blue dashed lines with ‘x’) during unfolding are shown for the wildtype. (**E**) The native state contacts (red dashed lines) for the residue at the mutation position (GLU-89) and the contacts broken (red dashed lines with ‘x’) during unfolding are shown for the mutant. The contacts broken are ranked in the order they were broken (numbers on lines). Only those contacts that share the same rank between the mutant and wildtype are considered for the calculation of ΔΔG.

To calculate ΔΔG, PSP-GNM considers both residue-residue interaction energies and entropies. While the residue–residue contact energies are obtained using the Miyazawa–Jernigan potential statistical potential, the MSF in distance between a pair of residues is a measure of their entropy. The difference between the energy and entropy terms of the mutant and wildtype proteins constitutes the ΔΔG calculated by PSP-GNM. Figure 1 provides an overview of PSP-GNM. Details of calculations and a more elaborate flow diagram are included in Materials and Methods.

We used five benchmark datasets from the literature in this study. **i.** The S2298 dataset includes 2298 forward mutations from 126 distinct proteins. It is by far the largest dataset available in the literature and is often used for training supervised methods. **ii.** The S350 dataset comprises 350 forward mutations spanning 67 proteins. We excluded one PDB ID and its corresponding mutation, as the PDB record was obsolete, resulting in 349 mutations across 66 proteins. Together, the S2298 and S350 datasets constitute the S2648 dataset, which contains 2648 mutations across 131 proteins. **iii.** The S611 dataset includes 611 forward and reverse mutations and is a subset of the S350 dataset. We excluded two mutations corresponding to an obsolete PDB record. **iv.** The S669 dataset includes 669 forward mutations across 94 distinct proteins. The proteins included in this dataset share less than 25% sequence identity with the proteins in the S2298 and S350 datasets. For this study, we also considered the reverse mutations corresponding to the forward mutations on this dataset. **v.** The Ssym+ dataset includes 704 mutations (352 forward and 352 reverse) across 371 proteins. This dataset is particularly useful for evaluating the antisymmetric property.

First, we identified the optimal parameters for which PSP-GNM shows maximum agreement with experimental ΔΔG. We used the S2298 dataset for this purpose. Additionally, we investigated the extent of agreement between the residue mean-squared fluctuations calculated by our method with the experimental temperature factors. Second, we evaluated the extent of agreement between PSP-GNM-calculated ΔΔG and experimental ΔΔG on the S2298, S350 and S611 datasets for different experimental conditions. Finally, we compared the performance of PSP-GNM with other state-of-the-art methods on the S350, S611, S669 and Ssym+ datasets.

### 2.1. Optimal Parameters for PSP-GNM

Two essential parameters regulating the native state protein dynamics captured by PSP-GNM are the cutoff distance (r_c_) between C-alpha atoms and the number of low-frequency modes (N_modes_) used to reconstruct the inverse Kirchhoff matrix. While r_c_ controls the number of interacting residue pairs in a protein, N_modes_ impacts the calculated MSF in distance between a pair of residues. Using the S2298 dataset, we identified values for these two parameters that maximized the agreement between the calculated and experimental ΔΔG. We chose the S2298 dataset because it provides a large and diverse set of mutations on which the parameters can be generalized. Furthermore, numerous existing methods have been trained using this dataset; the performance of PSP-GNM cannot be fairly compared with other methods on this dataset.

First, we set N_modes_ to 10 and identified the r_c_ in the range of 7Å–12Å that results in maximum correlation between the calculated and experimental ΔΔG. We observed the highest Pearson correlation coefficient of 0.39 for r_c_ = 9 Å (Table 1), which also had the highest proportion of mutations with a calculated ΔΔG (94%). As previously noted, PSP-GNM can only calculate a ΔΔG if there is at least one broken contact for the mutation position, both in the mutant and the wildtype. Then, we set r_c_ to 9 Å and observed the highest correlation of 0.4 for N_modes_ = 20 compared to 5, 10, 20, 30, 40 and 50 modes (Table 2). The correlation for N_modes_ = 10 was only marginally inferior (Pearson correlation 0.39), although we did observe that using 20 modes resulted in a higher proportion of mutations (95.4%) with a calculated ΔΔG. To choose between N_modes_ = 20 and N_modes_ = 10, we also considered evidence from the literature. Previously, it has been shown that using the first 10 low frequency modes is usually sufficient to capture the native state protein dynamics [40]. In addition, we have previously shown that the stability differences between wildtype and mutant structures can be well explained by using just the first 10 low frequency modes [33]. Consequently, we selected the parameters N_modes_ = 10 and r_cutoff_ = 9 Å and used these parameters throughout this study. PSP-GNM, however, does provide an option to run predictions using N_modes_ = 20.

Using the S2298 data, we also investigated the extent of agreement between the residue mean-squared fluctuations obtained using PSP-GNM and the experimental B-factors. We performed this comparison on the 126 wildtype proteins that were represented as C-alpha coarse-grained structures. Only 110 proteins were X-ray crystal structures and included B-factors. We observed a median correlation of 0.46 between the mean-squared fluctuations calculated by PSP-GNM and the experimental B-factors (Figure S1). We also note that this agreement was weaker than GNM (correlation 0.57) when using r_c_ = 9 Å and N_modes_ = 10 (Figure S1).

### 2.2. Predictive Performance of PSP-GNM on Three Datasets

We evaluated the extent of agreement between the PSP-GNM-calculated ΔΔG and the experimental ΔΔG on three datasets: S2298, S350 and S611. Specifically, we evaluated the performance under different experimental conditions. We also used the S2298 dataset to fit the regression line and obtain the scaling coefficients that were then used to scale the calculated ΔΔG.

#### 2.2.1. Performance on S2298 Dataset

**Figure 2:**
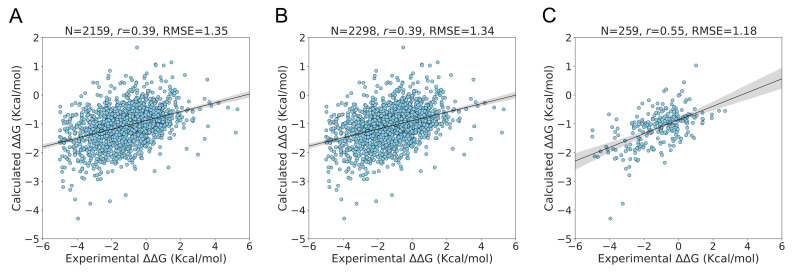
**Performance on S2298 dataset.** The extent of agreement between the experimental ΔΔG and the PSP-GNM-calculated ΔΔG is shown for (**A**) 2159 mutants having a PSP-GNM-calculated ΔΔG, (**B**) all 2298 mutants, (**C**) 259 mutants with experimental temperatures ranging from 24 °C to 26 °C and pH ranging from 6.8 to 7.2. The regression line with the 95% confidence interval (shaded gray) is shown for all three cases. The optimal agreement is seen for (**C**), with a Pearson correlation of 0.55 and an RMSE of 1.18 Kcal/mol. We used a theoretical ΔΔG value of 0 kcal/mol for cases without a PSP-GNM-calculated ΔΔG.

Of the 2298 mutations in the S2298 dataset, PSP-GNM-calculated ΔΔG could be obtained for 2159 mutations that had at least one broken contact involving the mutation position. For cases without a calculated ΔΔG, we assigned a theoretical ΔΔG value of 0 Kcal/mol. As we previously noted, using the selected parameters r_c_ = 9 Å and N_modes_ = 10 gave a Pearson correlation of 0.39 and an RMSE of 1.35 kcal/mol (Figure 2A). To scale the calculated ΔΔG, we used the equation of the regression line fit to the experimental ΔΔG. Upon inclusion of the 143 mutations without any PSP-GNM-calculated ΔΔG (by assigning them a theoretical ΔΔG of 0 kcal/mol), we did not observe any considerable change in the agreement to the experimental data (Figure 2B).

The distribution of the experimental temperatures and pH for the 2159 mutations with calculated ΔΔG indicated a peak around 25 °C and a pH close to 7 (Figure S2), suggesting that the experimental measurements were performed mostly around this temperature and pH. We then tested whether PSP-GNM showed better agreement with ΔΔG measurements performed around 25 °C and a pH of 7. Of the 2159 mutations, we identified 259 mutations with experimental temperatures ranging from 24 °C to 26 °C and pH ranging from 6.8 to 7.2 and observed an improved agreement (Pearson correlation = 0.55, RMSE = 1.18 Kcal/mol) with the experimental measurements (Figure 2C). We then asked: how does the agreement vary for mutations with experimental temperatures in the range of 24 °C–26 °C, but without any pH filter? How does the agreement vary for mutations with experimental pH of 6.8–7.2, but no temperature filter? Interestingly, we observed stronger agreement for mutations with experimental temperatures of 24 °C–26 °C (Pearson correlation = 0.45, RMSE = 1.31 Kcal/mol) compared to mutations with pH of 6.8–7.2 (Pearson correlation = 0.36, RMSE = 1.41 Kcal/mol) (Figure S3), suggesting a certain degree of bias towards measurements made at a particular temperature range.

#### 2.2.2. Performance on S350 Dataset

**Figure 3:**
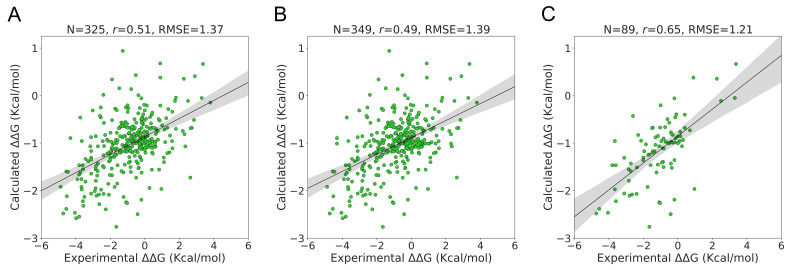
**Performance on S350 dataset.** Agreement between the experimental ΔΔG and the PSP-GNM-calculated ΔΔG is shown for (**A**) 325 mutants with a PSP-GNM-calculated ΔΔG, (**B**) all 349 mutants, (**C**) 89 mutants with experimental temperatures ranging from 24 °C to 26 °C and pH ranging from 6.8–7.2. The regression line with the 95% confidence interval (shaded gray) is shown for all three cases. The optimal agreement is seen for (**C**), with a Pearson correlation of 0.65 and an RMSE of 1.21 Kcal/mol.

The S350 dataset included 349 mutations from 66 unique PDB identifiers. On this dataset, ΔΔG could be calculated with PSP-GNM for 325 wildtype–mutant pairs that exhibited at least one broken contact involving the mutation position during partial unfolding. We observed a Pearson correlation of 0.51 and an RMSE of 1.37 Kcal/mol for these 325 mutations (Figure 3A). When the remaining 24 wildtype–mutant pairs were included by assigning them a ΔΔG value of 0 kcal/mol, we observed a decrease in the Pearson correlation to 0.49 and an increase in the RMSE to 1.39 Kcal/mol (Figure 3B).

The experimental temperature and pH for the 318 mutants with calculated ΔΔG show a similar distribution as that of the S2298 dataset, i.e., a peak around 25 °C and pH 7 (Figure S4). We observed a considerably stronger agreement with the experimental ΔΔG when considering a subset of 89 mutations with experimental temperatures from 24 °C to 26 °C and pH from 6.8 to 7.2 than when considering all the data (Figure 3C). The associated Pearson correlation and RMSE were 0.65 and 1.21 Kcal/mol, respectively. The agreement was stronger when we considered a temperature range of 24 °C–26 °C without any pH cutoff (Pearson correlation = 0.57, RMSE = 1.39 Kcal/mol) than when we considered a pH range of 6.8–7.2 without any temperature cutoff (Pearson correlation = 0.5, RMSE = 1.36 Kcal/mol) (Figure S5).

Additionally, we investigated the extent to which PSP-GNM satisfied the antisymmetric property of forward and reverse mutations using the S350 dataset. Ideally, the forward and reverse mutations should be antisymmetric in terms of their ΔΔG, i.e., ΔΔG*_forward_*= −ΔΔG*_reverse_*. We observed a strong negative correlation of −1 between the calculated ΔΔG*_forward_* and ΔΔG*_reverse_* (Figure S6), suggesting that the calculations using PSP-GNM satisfy the expected antisymmetric property.

#### 2.2.3. Performance on S611 Dataset

In contrast to the S350 and S2298 datasets, the S611 dataset evaluated the performance of PSP-GNM on both forward and reverse mutations. The S611 was created using a subset of data from the S350 dataset and comprises 609 mutations from 66 unique PDB identifiers. PSP-GNM could calculate ΔΔG for 564 wildtype–mutant pairs, as the remaining 45 pairs did not involve a contact break for the residue at the mutation position.

**Figure 4:**
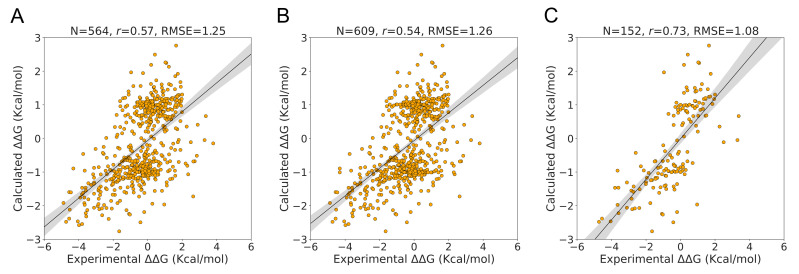
**Performance on S611 dataset.** Agreement between the experimental ΔΔG and the PSP-GNM-calculated ΔΔG is shown for (**A**) 564 mutants with a PSP-GNM-calculated ΔΔG, (**B**) all 609 mutants, (**C**) 152 mutants with experimental temperatures ranging from 24 °C to 26 °C and pH ranging from 6.8 to 7.2. The regression line with the 95% confidence interval (shaded gray) is shown for all three cases. The optimal agreement is seen for (**C**), with a Pearson correlation of 0.73 and an RMSE of 1.08 Kcal/mol.

When considering the calculated and experimental ΔΔG for the 564 mutations, we observed a correlation of 0.57 and an RMSE of 1.25Kcal/mol (Figure 4A), demonstrating stronger agreement than was observed for the 325 forward mutations in the S350 dataset. As seen in Figure 4B, when we included the 45 mutations without PSP-GNM-calculated ΔΔG, we observed a decrease in the agreement (Pearson correlation = 0.54, RMSE = 1.26 kcal/mol). However, when we limited the analysis to only those mutations with experimental temperatures of 24 °C–26 °C and pH of 6.8–7.2, we noted a considerable improvement to 0.73 in the correlation and a reduction to 1.08 Kcal/mol in the RMSE (Figure 4C). Interestingly, we did not observe considerable differences in correlations for measurements made between 24 °C and 26 °C without any pH filter (Pearson correlation = 0.61) and those made near neutral pH (6.8–7.2) without a temperature filter (Pearson correlation = 0.62) (Figure S7).

### 2.3. Performance Comparisons with Existing Methods on Four Datasets

We compared the performance of PSP-GNM with other state-of-the-art methods on four datasets: S350, S611, S669 and Ssym+. On the S350 and S611 datasets, we assessed the performance against nine existing methods: Dynamut2 [25], Dynamut [24], MCSM [22], MUPro [41], ENCOM [42], DUET [43], SDM [17], I-Mutant2 [19] and Maestro [23]. While ENCOM and SDM are unsupervised methods, the remaining methods use machine learning for predicting ΔΔG. The recently developed Dynamut2 approach also considers the above methods for comparison, and we considered them for this study because they are a good mixture of supervised and unsupervised methods. On the S669 and Ssym+ datasets, we reported performance comparisons against a broader set of 20 methods, including 8 methods considered for the S350 and S611 datasets.

For each dataset, we compared across methods by considering two groups of mutations: *Group 1*, consisting only of those mutations that had a PSP-GNM-calculated ΔΔG, and *Group 2*, consisting of the mutations in Group 1 with an experimental temperature ranging from 24 °C to 26 °C and pH ranging from 6.8 to 7.2.

#### 2.3.1. S350 Dataset

In Table 3, we report the comparisons made for Group 1 across the 10 methods using data obtained from Rodrigues et al. [25]. Of the total 349 mutations in the S350 dataset, the comparisons were made using the 325 wildtype–mutant pairs with a ΔΔG calculated. PSP-GNM ranked sixth, with a correlation of 0.51 and a RMSE of 1.37 Kcal/mol, while DUET showed the best performance, with a correlation of 0.67 and an RMSE of 1.16 Kcal/mol. When we performed the same comparison on Group 2 mutants that had a total number of 89 mutants, PSP-GNM ranked fifth, showing a correlation of 0.65 and an RMSE of 1.21 Kcal/mol, while Dynamut2 and DUET topped the list with a correlation of 0.73 and an RMSE of 1.05 kcal/mol (Table 4).

#### 2.3.2. S611 Dataset

In Table 5, we report the comparisons for Group 1 mutants from the S611 dataset using data obtained from Rodrigues et al. [25]. The total number of mutations taken into consideration in this group was 564. Remarkably, we observed that PSP-GNM performed better than most of the other methods, ranking second with a correlation of 0.57 and an RMSE of 1.54 Kcal/mol. The best performance was seen for Dynamut2 (correlation = 0.68, RMSE = 1.08 Kcal/mol). There were 152 mutants that were considered in Group 2 (Table 6). PSP-GNM demonstrated strong agreement with the experimental ΔΔG by ranking second with a correlation of 0.73 and an RMSE of 1.08 Kcal/mol. Overall, the best performance for this group was seen for Dynamut2 (correlation = 0.79 and RMSE = 0.95 Kcal/mol).

#### 2.3.3. S669 Dataset

The S669 dataset was created as part of a recent study by Pancotti et al. [44] and has <25% sequence identity with proteins included in the S2298 dataset. Consequently, it provides an unbiased platform to compare those methods that were trained using the S2298 dataset, or even the larger S2648 dataset. On this dataset, we compared the performance of PSP-GNM with 20 existing methods that were studied by Pancotti et al. [44]. A detailed description of each method can be found in the original article.

In Table 7, we report the performance for these 20 methods over 584 mutations (forward and reverse) with a PSP-GNM-calculated ΔΔG. We also include information on bias and antisymmetry for each method, using data from Pancotti et al. [44]. The table is sorted by Pearson correlation for the Forward + Reverse category. PSP-GNM showed remarkably better performance compared to 17 existing methods when considering both forward and reverse mutations (Pearson correlation = 0.61 and RMSE = 1.57 Kcal/mol). It had 0 bias and a perfect antisymmetry of −1. The best performance on the Forward + Reverse category was seen for PremPS and ACDC-NN. PSP-GNM performed better than 11 methods for the reverse mutations, while performing better than only 4 methods for the forward mutations.

When we compared performance across all 669 mutations (by using a theoretical ΔΔG value of 0 Kcal/mol for cases without a ΔΔG), PSP-GNM ranked fifth, with a Pearson correlation of 0.59 for the Forward + Reverse mutants (Table S2). It showed better performance than nine other methods for the reverse mutants but only three methods for the forward mutants. Interestingly, we did not observe any performance gain when considering only those mutations with experimental temperatures ranging from 24 °C to 26 °C and pH ranging from 6.8 to 7.2 (Table S3).

#### 2.3.4. Ssym+ Dataset

The Ssym+ dataset is an extension of the Ssym dataset and includes 352 forward and 352 reverse mutations [13,45]. Importantly, each pair of forward-reverse mutants has separate experimentally resolved PDB structures for the forward and reverse mutant. Consequently, it serves as an excellent dataset to check the performance for forward and reverse mutations and test for antisymmetry and bias. Like the S669 data, we compared the performance of PSP-GNM against 20 existing methods for a subset of 341 mutations with a PSP-GNM-calculated ΔΔG. The data were obtained from Pancotti et al. [44]. PSP-GNM reported near-perfect antisymmetry, with a Pearson correlation of −0.97 and 0 bias (Table 8, Figure S8). When considering both forward and reverse mutations, PSP-GNM ranked seventh with a correlation of 0.64, while PremPS showed the best performance with a correlation of 0.85 (Table 8). Considering the reverse mutations, PSP-GNM showed better performance than 10 other methods, with a correlation of 0.37. All methods outperformed PSP-GNM on the forward mutation dataset. When considering all 352 mutations, PSP-GNM ranked seventh on the Forward + Reverse category, with a correlation of 0.63. It exhibited antisymmetry -0.96 and 0 bias (Table S4).

We then considered mutations with experimental temperatures ranging from 24 °C to 26 °C and pH ranging from 6.8 to 7.2. Only 10 mutations fell in this category, making it a very small sample size. Therefore, we considered a broader temperature range (20 °C–30 °C), while keeping the pH range the same. We observed improvement in the agreement for both the forward mutants (Pearson correlation = 0.74) and reverse mutants (Pearson correlation = 0.71), while noticing a drop in the correlation for the Forward + Reverse category (Pearson correlation = 0.52). PSP-GNM ranked seventh in terms of its performance in the Forward + Reverse category (Table 9).

## 3. Discussions

The fundamental assumption underlying PSP-GNM is that during unfolding, the contacts broken for the mutation position differ between the wildtype and mutant forms of a protein. When contacts are ranked in the order in which they break with respect to the mutation position, and the contacts that share the same rank in the mutant and wildtype are considered, the difference in their free energies correlates with the experimental ΔΔG, as demonstrated in this study. In this context, the free energy is the interaction energy between a pair of residues given by the Miyazawa–Jernigan statistical potential, and the entropy is the mean-squared fluctuation in distance between a pair of residues. The choice of restricting the calculations to only a subset of contacts sharing the same rank is arguable. However, an initial pilot test performed as part of this study revealed that including all the broken contacts (irrespective of their ranks) resulted in poorer agreement with the experimental ΔΔG when compared to the present scheme. A detailed examination of various possible functions using knowledge of contacts broken to quantify ΔΔG is, however, beyond the scope of the current work, and is a subject for future discussion.

In this study, we used a weighted Gaussian network model that used Hookean springs of variable stiffness to simulate specific interactions between a pair of residues. An approach of weighting the Hookean springs between interacting pairs of residues based on their identity and chemical nature has been previously shown to improve the ability of elastic network models to capture the near-native protein dynamics [51]. Through PSP-GNM, we demonstrated that despite maintaining a good agreement with the experimental B-factors, such weighted formulations of elastic network models can also discriminate between the native and mutant forms of a protein. The partial unfolding approach used in our study is a modification of a previous method described by Su et al. [34] that was able to successfully predict the order of contact break events. Additionally, to verify the extent of concordance between PSP-GNM and the original GNM, we randomly picked two proteins (PDB IDs 1AKY and 1CUN, chain A) from our dataset and obtained the residue–residue cross-correlation heat maps for the wildtype using both methods (Figure S9). A visual inspection suggests close correspondence between the cross-correlation maps obtained from the two methods. Since PSP-GNM is an implementation of GNM with weighted contacts, some difference in the cross-correlation maps is expected. Despite the differences, the expected high correlations between contiguous residues of secondary structures are still consistent with GNM.

A major limitation of PSP-GNM is its inability to provide a calculated ΔΔG if no contacts involving the residue at the mutation position are broken in the wildtype and/or in the mutant. To further understand why no contacts are broken for certain mutations, we randomly selected two wildtype–mutant protein pairs from the S350 dataset and observed that the residue contacts for the mutation positions were primarily within the same secondary structure elements (Figure S10). Residue–residue contacts within secondary structures are energetically stronger and less easily broken than tertiary contacts, possibly explaining why they were not broken during partial unfolding. To address this limitation, we re-defined partial folding by increasing the cutoff to 60%. That is, we considered a protein to be partially unfolded if 60% of the native state contacts were broken. However, we observed that the model became unstable by generating more than one zero eigen values upon eigen decomposition of the Kirchhoff matrix, suggesting a sparse contact matrix. Consequently, we assigned a theoretical ΔΔG value of 0 for such cases. Based on the results presented for the S2298 (Figure 2B), S350 (Figure 3B) and S611 (Figure 4B) datasets, we do not observe a considerable reduction in the agreement with experimental ΔΔG when assigning a PSP-GNM-calculated ΔΔG value of 0 for such cases.

The performance comparisons made on the datasets in this study indicate superior performance of many existing methods compared to PSP-GNM. In this context, a simple investigation of the extent of sequence similarity of proteins in each of the test datasets (S350, S669 and Ssym+) with the proteins in the S2298 dataset reveals that most datasets showed considerable sequence identity (>30%) with the proteins in the S2298 dataset (Tables S5–S8). The only exception was the S669 dataset, which had only four proteins with >25% sequence identity. Because most of the methods evaluated in our study are supervised and have mostly been trained on the S2298 dataset or its variants, a superior performance on the S350, S611 and Ssym+ datasets is not unexpected. On the S669 dataset, PSP-GNM outperformed many of the existing state-of-the-art methods, specifically when considering the reverse mutations.

Three key observations were made in our study. First, we observed variation in the extent of agreement between the calculated ΔΔG and the experimental ΔΔG for different experimental temperatures and pH. We demonstrated that the agreement was strongest for mutants with experimental temperatures close to 25 °C and near-neutral pH. For the S350, S611 and Ssym+ datasets, we observed that the correlation was higher for PSP-GNM than for most other methods. Previous studies have also used higher weights for measurements made in the same temperature and pH conditions [11,52]. One must note that the ProTherm database not only reports ΔΔG values based on thermal denaturation experiments, but also ΔΔG values from chemical denaturation experiments. In the current study, data from both experiments are treated alike, and it is assumed that the same scheme for simulating unfolding may be used. It is also to be noted that PSP-GNM currently does not consider the experimental temperature to weight the interactions between residues. Based on the observed temperature dependence, however, using experimental temperature as a parameter for weighing residue–residue interactions could further improve the extent of agreement with experimental ΔΔG. Second, considering just the contacts broken with the mutation position and their order is key to the reasonably good correlation observed. It strongly suggests the importance of geometric and energetic changes localized at the mutation site in predicting the overall change in thermodynamic stability of proteins. Finally, the assessment for antisymmetry made on the Ssym+ dataset demonstrates the ability of PSP-GNM to maintain the antisymmetric relationship between forward and reverse mutations, which is indeed a hallmark of this approach.

## 4. Materials and Methods

### 4.1. Datasets

We used five benchmark datasets from the literature. The composition of each dataset is described in Table 10.

Of the five datasets, we used the S2298 dataset to obtain the optimal parameters for PSP-GNM. We chose the S2298 dataset for this purpose because it includes a diverse set of mutations. Furthermore, most of the existing supervised methods have used this dataset for training. Therefore, predictive performances cannot be fairly compared on this dataset. Each dataset contains information on non-synonymous point mutations and the experimentally measured ΔΔG for different proteins. We excluded mutations that showed mismatches for the wildtype residue in the PDB file and those with obsolete PDB entries. A larger fraction of mutations in each dataset are destabilizing mutations, i.e., ΔΔG < 0 kcal/mol. It is to be noted that the Ssym+ dataset comprises separate PDB structures for forward and reverse mutants and has previously been used to evaluate the antisymmetry and bias for different methods. In this context, if x-N-y denotes a forward mutation from amino acid x to amino acid y at position N and has ΔΔG = δ Kcal/mol, then the reverse mutation (y-N-x) is antisymmetric to the forward mutation and has ΔΔG = −δ Kcal/mol.

### 4.2. PSP-GNM Algorithm

We represented the wildtype and mutant protein structures as coarse-grained systems where each residue was represented by its alpha carbon. Additionally, we represented the mutant protein structures by replacing the wildtype residues with their respective mutant residues at the alpha carbon position. We then took a similar approach using GNM to simulate protein unfolding and identify the sequence of residue–residue contacts broken during unfolding, as previously described by Su et al. [34]. Specifically, Su et al. demonstrated protein unfolding using GNM for two proteins (Barnase and Chymotrypsin inhibitor) and observed significant agreement in the sequence of unfolding events with atomic molecular dynamics and Monte Carlo simulations. Like their approach, we also used a weighted GNM where the strength of springs between interacting residues varies with the interacting residue pair.

In GNM, the protein residues are represented by alpha carbons, and residue pairs within a cutoff distance r_c_ are hypothetically connected with Hookean springs of force constant γ=1. In PSP-GNM, we weight the interactions between residues by their corresponding Miyazawa–Jernigan (MJ) contact potential [36], which is obtained from the AAindex database [53] using the identifier MIYS960101 (upper half of the original MJ potential table).Specifically, the contact potential for a residue pair is converted into its Boltzmann weight (by simply taking its exponential) and is then used to weight the interaction between the pair of residues. The potential for PSP-GNM is given as

(1) $$V=\frac{1}{2}\gamma \left[\overset{N}{\underset{i,j}{\sum }}∆R_{i}\Gamma_{ij}∆R_{j}\right]$$

where $∆R_{i}$ is the fluctuation vector for residue *i* and $∆R_{j}$ is the fluctuation vector for residue *j.* The fluctuation vector defines the instantaneous fluctuation of a residue from its equilibrium position. The force constant γ is weighted for each interacting residue pair depending on the type of interaction. $\Gamma_{ij}$ is the *ij*th element in the Kirchhoff matrix of inter-residue contacts and is given as follows.

(2) $$\Gamma_{ij}=\{\begin{array}{c} -\exp\left(-\left(\min\left(MJ\right)-1\right)\right),\;if\left|i-j\right|=1\;\\ -\exp\left(MJ_{ij}\right),\;if\left|i-j\right|>1\;and\;d_{ij}\;\leq r_{c}\\ \;0,\;\;if\left|i-j\right|>1\;and\;d_{ij}>r_{c}\\ -\overset{N}{\underset{i,\;j\neq i}{\sum }}\Gamma_{ij},\;if\;i=j \end{array}$$

In Equation (2), $MJ_{ij}$ is the contact energy between residue *i* and residue *j* as given in the MJ potential matrix, $d_{ij}$ is the distance between the alpha carbons of residues *i* and *j* and $r_{c}$ is the distance cutoff for interacting residues. Stiffer springs were used to simulate the stronger covalent interactions (|i−j|=1) between adjacent residue pairs by using the minimum value in the MJ potential matrix minus one.

To simulate unfolding, we iteratively identified residue–residue contacts with the highest mean-squared fluctuations (MSF) in their distance [34,54] and then removed the contacts between these residues. The MSF in distance between residues *i* and *j*, also referred to as internal distance change, is the average fluctuation in their internal distance. A residue pair with strong interaction energy would have lower MSF in their distance compared to a residue pair with weak interaction energy. The MSF in distance between residue pairs *i* and *j* is calculated as

(3) $$<\left(∆R_{ij}^{2}\right)>=\frac{3K_{B}T}{\gamma }(\Gamma_{ii}^{-1}+\Gamma_{jj}^{-1}-2\Gamma_{ij}^{-1})$$

where $K_{B}$ is the Boltzmann constant and *T* is the absolute temperature in Kelvin. $\Gamma_{}^{-1}$ is the pseudo-inverse of the Kirchhoff matrix and is given as

(4) $$\Gamma_{}^{-1}=\left(\frac{3K_{B}T}{\gamma }\right)\overset{N-1}{\underset{k=1}{\sum }}\lambda_{k}^{-1}V_{k}V_{k}^{T}$$

As the determinant of the Kirchhoff matrix is zero, its inverse cannot be directly calculated. Instead, we calculated the pseudo-inverse using the *N*−1 non-zero eigen values and their corresponding eigen vectors. In the above equation, $\lambda_{k}$ is the *k*th eigen value, $V_{k}$ is the *k*th eigen vector associated with $\lambda_{k}$ and $V_{k}^{T}$ is its transpose.

**Figure 5:**
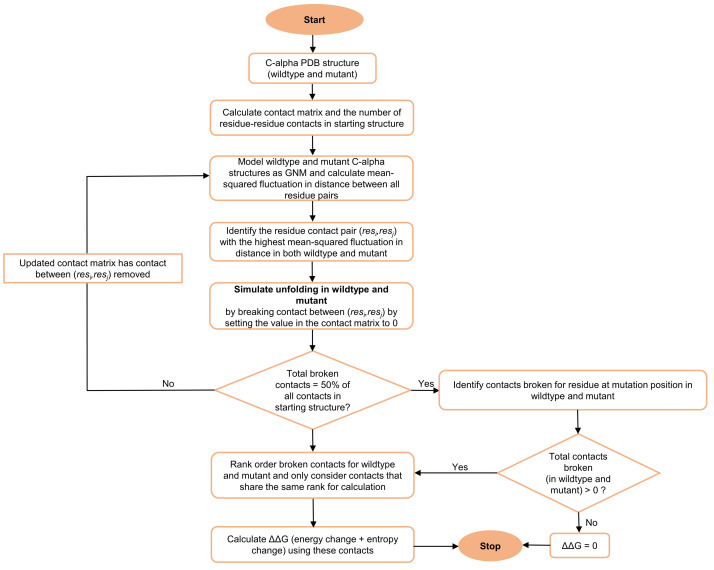
**PSP-GNM workflow.** The steps involved in PSP-GNM are outlined. The input is a coarse-grained protein structure that includes only the residue C-alpha atoms. The protein is then partially unfolded by sequentially identifying the residue contact pairs with the highest mean-squared fluctuations in their distance and then removing the contacts between these residue pairs. The process is repeated until 50% of native contacts are broken. These steps are repeated separately for the wildtype and mutant proteins. The contacts broken for the mutation position are then identified in the wildtype and mutant forms and ranked in the order they were broken. The subset of contacts that share the same rank in the wildtype and mutant are then used to calculate ΔΔG.

The steps in our algorithm are outlined in Figure 5. Given the wildtype or mutant C-alpha PDB structure, we first identified the total number of residue–residue contacts in the starting structure. A contact pair is a residue pair whose C-alpha atoms are within 9 Å. We then calculated the MSF in distance between all pairs of residues by using only the 10 slowest non-zero modes to re-construct the $\Gamma_{}^{-1}$. The above values for the r_c_ (C-alpha distance cutoff) and number of modes parameters were used, as they resulted in maximum agreement between the experimental ΔΔG and PSP-GNM-calculated ΔΔG on the S2298 dataset. The contact pair with the largest MSF in distance was then identified, the contact between the residue pair was broken, and the contact matrix was updated to reflect this contact break. The updated contact matrix was then used in the next iteration to identify the contact pair with the largest MSF and highest likelihood of contact break. We repeated this scheme of breaking contacts based on the largest MSF until 50% of all contacts in the starting structure were broken, resembling a partially unfolded protein, or until the model became unstable, resulting in more than one 0-eigen values upon eigen decomposition of the Kirchhoff matrix.

### 4.3. Calculation of ΔΔG Using PSP-GNM

PSP-GNM assumes a given mutant protein differs from its corresponding wildtype by only one amino acid. That is, the current implementation of PSP-GNM can be used to calculate ΔΔG given a mutant structure with a single amino acid change compared to its wildtype. For a pair of wildtype and mutant proteins, the contacts broken with the residue at the mutation position during partial unfolding are serially ranked (starting with a rank of 1) in the order in which they were broken. The underlying assumption is that there is at least a single contact involving the residue at mutation position that is broken during the partial unfolding of the mutant and wildtype structures. Then, we only considered the set of broken contacts that shared the same rank in the mutant and wildtype. ΔΔG was then calculated as

(5) $$∆∆G_{PSP\_GNM}=-\left(\overset{N}{\underset{j=1}{\sum }}(∆G_{i,j}^{mut}-∆G_{i,j}^{wt})-\overset{N}{\underset{j=1}{\sum }}(∆S_{i,j}^{mut}-∆S_{i,j}^{wt})\right)$$

(6) $$∆∆G_{PSP\_GNM}=-\left(\overset{N}{\underset{j=1}{\sum }}(MJ_{i,j}^{mut}-MJ_{i,j}^{wt})-\overset{N}{\underset{j=1}{\sum }}(MSF_{i,j}^{mut}-MSF_{i,j}^{wt})\right)$$

In the above, $\overset{N}{\underset{j=1}{\sum }}(∆G_{i,j}^{mut}-∆G_{i,j}^{wt})$ is the total difference in the interaction energies of the mutant and wildtype; $\overset{N}{\underset{j=1}{\sum }}(∆S_{i,j}^{mut}-∆S_{i,j}^{wt})$ is the difference in entropy. $MJ_{i,j}^{mut}$ and $MJ_{i,j}^{wt}$ are the Miyazawa–Jernigan interaction energies between the residue at mutation position *i* and the residue with rank *j* with which contact was broken in the mutant and wildtype proteins, respectively. The MSF in distance is considered as a measure of entropy. $MSF_{i,j}^{mut}$ and $MSF_{i,j}^{wt}$ are the mean-squared fluctuations between the residue at mutation position *i* and the residue with rank *j* with which contact was broken in the mutant and wildtype proteins, respectively. Re-arranging the terms, Equation (5) can be re-written as

(7) $$∆∆G_{PSP\_GNM}=-\left((\overset{N}{\underset{j=1}{\sum }}∆G_{i,j}^{mut}-\overset{N}{\underset{j=1}{\sum }}∆G_{i,j}^{wt})-\left(\overset{N}{\underset{j=1}{\sum }}∆S_{i,j}^{mut}-\overset{N}{\underset{j=1}{\sum }}∆S_{i,j}^{wt}\right)\right)$$

(8) $$∆∆G_{PSP\_GNM}=-\left(\left(\overset{N}{\underset{j=1}{\sum }}∆G_{i,j}^{mut}-\overset{N}{\underset{j=1}{\sum }}∆S_{i,j}^{mut}\right)-\left(\overset{N}{\underset{j=1}{\sum }}∆G_{i,j}^{wt}-\overset{N}{\underset{j=1}{\sum }}∆S_{i,j}^{wt}\right)\right)$$

A strong underlying assumption of PSP-GNM is that the local unfolding energetics of the mutation position have considerable association with the unfolding free energy change. Consistent with this assumption and based on Equations (5) and (6), the calculations for ΔΔG can only be made if there exists at least one broken contact involving the mutation position in both the mutant and the wildtype. For mutations that do not exhibit a broken contact involving the mutation position, the wildtype and mutant forms are less likely to differ from each other in their partially unfolded states with respect to the mutation position. Hence, we assigned a theoretical ΔΔG value of 0. The calculated ΔΔG was re-scaled using the equation for the regression line obtained upon fitting the calculated and experimental ΔΔG on the S2298 dataset.

### 4.4. Calculation of ΔΔG for Reverse Mutations

To perform calculations for reverse mutations (ΔΔG*_mutant_*→ΔΔG*_wildtype_*), we simply switched the wildtype and the mutant structures. Then, we followed the same steps as described above to calculate ΔΔG.

### 4.5. Evaluation Metrics

To estimate the extent of agreement between calculated ΔΔG and experimental ΔΔG, we used the Pearson correlation coefficient and root mean squared error, as defined below.

(9) $$PCC\left(∆∆G_{PSP\_GNM},\;∆∆G_{exp}\right)=\frac{Covariance\left(∆∆G_{PSP\_GNM},\;∆∆G_{exp}\right)}{\sigma_{∆∆G_{PSP\_GNM}}\sigma_{∆∆G_{exp}}}$$

(10) $$RMSE\left(∆∆G_{PSP\_GNM},\;∆∆G_{exp}\right)=\sqrt{\frac{\sum_{i}{\left(∆∆G_{PSP\_GNM}^{i}-∆∆G_{exp}^{i}\right)}^{2}}{N}}$$

The extent of antisymmetry between the calculated ΔΔG for forward and reverse mutants was evaluated using the Pearson correlation coefficient, as described previously by Pancotti et al. [44].

(11) $$Antisymmetry_{\left(forward,\;reverse\right)}=PCC\left(∆∆G_{PSP\_GNM}^{forward},\;∆∆G_{PSP\_GNM}^{reverse}\right)\;$$

We evaluated the bias towards any class of mutation (forward/reverse) using the bias score (δ) [45].

(12) $$Bias\;score\left(\delta \right)=\frac{\sum_{i=1}^{N}(∆∆G_{i}^{forward},\;∆∆G_{i}^{reverse})}{2N}$$

## 5. Conclusions

Understanding the thermodynamic stability changes induced by point mutations in proteins facilitates an understanding of the role of mutant proteins in various diseases. Numerous machine-learning-based methods have been developed to predict ΔΔG associated with point mutations. Many of these methods can predict the effect of multiple point mutations. In this study, we presented PSP-GNM, a novel approach utilizing the knowledge of intrinsic protein dynamics to quantify the thermodynamic changes associated with single amino acid substitutions in proteins. PSP-GNM differs from many of the existing methods in the respect that it does not use a machine learning approach. Through PSP-GNM, we have introduced a formulation of GNM in which interacting residues are weighted by their interaction energies obtained from the Miyazawa–Jernigan statistical potential. PSP-GNM utilizes the knowledge of putative contacts broken during partial protein unfolding to estimate ΔΔG. Although there exist several methods that can predict ΔΔG simply by using the protein sequence, PSP-GNM relies on protein structure to calculate ΔΔG. Additionally, the present implementation of PSP-GNM can only calculate the ΔΔG for single amino acid changes, i.e., for cases where the wildtype and mutant sequences differ by only a single amino acid. Our method also highlights the role of near-native protein dynamics in predicting changes to thermodynamic stability. Most importantly, the superior performance of PSP-GNM compared to other methods incorporating protein dynamics information (e.g., ENCOM) suggests the importance of including information on local unfolding and contact changes to predict stability changes upon mutations.

## Figures and Tables

**Table 1:** Predictive performance of PSP-GNM for different distance cutoffs evaluated on the S2298 dataset. For all calculations, only the top 10 low-frequency modes were used. % Predictions is the percentage of mutations in the S2298 dataset with a calculated ΔΔG. The best performance is shown in bold.

Number of Modes	Distance Cutoff (Å)	Pearson Correlation	RMSE (Kcal/mol)	% Predictions
**10**	**9**	**0.39**	**1.35**	**94**
10	11	0.39	1.34	91.7
10	12	0.38	1.35	93.7
10	7	0.38	1.36	91.4
10	10	0.37	1.36	94.3
10	8	0.37	1.36	93.3

**Table 2:** Predictive performance of PSP-GNM for different low-frequency modes evaluated on the S2298 dataset. For all calculations, the distance cutoff was set to 9 Å. % Predictions is the percentage of mutations in the S2298 dataset with a calculated ΔΔG. The best performance is shown in bold.

Number of Modes	Distance Cutoff (Å)	Pearson Correlation	RMSE (Kcal/mol)	% Predictions
20	9	0.4	1.34	95.4
**10**	**9**	**0.39**	**1.35**	**94**
40	9	0.39	1.35	94.6
5	9	0.39	1.36	88.4
50	9	0.39	1.36	93.9
30	9	0.38	1.35	95.1

**Table 3:** Performance on the S350 dataset for a subset of 325 mutations with a PSP-GNM-calculated ΔΔG. The 325 mutations had at least one contact broken, both in the wildtype and mutant forms, for the residue at the mutation position when simulating unfolding with PSP-GNM. The performance for PSP-GNM is shown in bold.

Method	Pearson Correlation	RMSE (Kcal/mol)
DUET	0.67	1.16
MCSM	0.66	1.19
Dynamut2	0.65	1.19
IMutant	0.54	1.32
Maestro	0.57	1.70
**PSP-GNM**	**0.51**	**1.37**
SDM	0.48	1.91
Dynamut	0.39	1.79
MUPro	0.18	1.63
ENCOM	0.15	2.23

**Table 4:** Performance on the S350 dataset for a subset of 89 mutations with a PSP-GNM-calculated ΔΔG and with experimental temperatures ranging from 24 °C to 26 °C and pH ranging from 6.8 to 7.2. The 89 mutations had at least one contact broken, both in the wildtype and mutant forms, for the residue at the mutation position when simulating unfolding with PSP-GNM. The performance for PSP-GNM is shown in bold.

Method	Pearson Correlation	RMSE (Kcal/mol)
Dynamut2	0.73	1.05
DUET	0.73	1.05
MCSM	0.71	1.08
Maestro	0.68	1.72
**PSP-GNM**	**0.65**	**1.21**
SDM	0.63	1.68
Dynamut	0.55	1.7
ENCOM	0.54	1.56
IMutant	0.49	1.33
MUPro	0.23	1.65

**Table 5:** Performance on S611 dataset for a subset of 564 mutations with a PSP-GNM-calculated ΔΔG. The 564 mutations had at least one contact broken, both in the wildtype and mutant forms, for the residue at the mutation position when simulating unfolding with PSP-GNM. The performance for PSP-GNM is shown in bold.

Method	Pearson Correlation	RMSE (Kcal/mol)
Dynamut2	0.68	1.08
**PSP-GNM**	**0.57**	**1.25**
DUET	0.49	1.42
MCSM	0.46	1.45
Maestro	0.38	1.45
SDM	0.36	1.96
IMutant	0.34	1.5
Dynamut	0.26	1.53
MUPro	0.15	1.69
ENCOM	0.14	1.81

**Table 6:** Performance on the S611 dataset for a subset of 152 mutations with a PSP-GNM-calculated ΔΔG and with experimental temperatures ranging from 24 °C to 26 °C and pH ranging from 6.8 to 7.2. The 152 mutations had at least one contact broken, both in the wildtype and mutant forms, for the residue at the mutation position when simulating unfolding with PSP-GNM. The performance for PSP-GNM is shown in bold.

Method	Pearson Correlation	RMSE (Kcal/mol)
Dynamut2	0.79	0.95
**PSP-GNM**	**0.73**	**1.08**
DUET	0.69	1.25
MCSM	0.65	1.34
SDM	0.6	1.76
ENCOM	0.6	1.32
Dynamut	0.51	1.41
Maestro	0.45	1.48
IMutant	0.32	1.62
MUPro	0.31	1.85

**Table 7:** Performance comparison on the S669 dataset for a subset of 584 mutations with a PSP-GNM-calculated ΔΔG. The table is sorted by Pearson correlation in the Forward + Reverse category. The performance for PSP-GNM is shown in bold.

Method	Forward		Reverse		Forward + Reverse		Bias/Antisymmetry	
	Pearson Correlation	RMSE (Kcal/mol)	Pearson Correlation	RMSE (Kcal/mol)	Pearson Correlation	RMSE (Kcal/mol)	Bias	Antisymmetry
PremPS [26]	0.4	1.57	0.41	1.56	0.62	1.56	0.09	−0.81
ACDC-NN [45]	0.45	1.56	0.44	1.57	0.62	1.56	−0.02	−0.98
INPS-Seq [46]	0.41	1.59	0.41	1.6	0.61	1.6	0	−1
**PSP-GNM**	**0.36**	**1.57**	**0.36**	**1.57**	**0.61**	**1.57**	**0**	**−1**
ACDC-NN-Seq [27]	0.41	1.6	0.41	1.6	0.59	1.6	0	−1
DDGun3D [18]	0.42	1.67	0.4	1.69	0.57	1.68	−0.04	−0.96
DDGun [18]	0.39	1.8	0.37	1.83	0.56	1.82	−0.06	−0.96
INPS3D [46]	0.42	1.56	0.31	1.85	0.55	1.71	−0.39	−0.52
Dynamut [24]	0.41	1.66	0.35	1.76	0.51	1.71	−0.07	−0.59
ThermoNet [28]	0.37	1.69	0.37	1.73	0.51	1.71	−0.05	−0.85
PopMusic [47]	0.41	1.56	0.27	2.17	0.47	1.89	−0.72	−0.36
MAESTRO [48]	0.48	1.51	0.21	2.18	0.45	1.88	−0.61	−0.26
DUET [43]	0.41	1.57	0.25	2.24	0.43	1.93	−0.7	−0.15
mCSM [22]	0.37	1.58	0.25	2.4	0.39	2.03	−0.89	−0.1
I-Mutant3.0-Seq [19]	0.34	1.6	0.21	2.32	0.38	1.99	−0.77	−0.46
Dynamut2 [25] *	0.34	1.58	0.17	2.16	0.36	1.9	−0.64	0.03
I-Mutant3.0 [19]	0.35	1.59	0.16	2.42	0.33	2.05	−0.83	−0.08
MuPro [41]	0.24	1.66	0.21	2.47	0.33	2.11	−0.96	−0.31
SDM [17]	0.4	1.74	0.14	2.25	0.32	2.01	−0.42	−0.4
FoldX [49]	0.21	2.41	0.22	2.6	0.3	2.51	−0.34	−0.18
SAAFEC-Seq [50]	0.35	1.59	0	2.5	0.27	2.09	−0.85	−0.03

* For Dynamut2, the performance metrics correspond to all the 669 mutations.

**Table 8:** Performance comparison on the Ssym+ dataset for a subset of 341 mutations with a PSP-GNM-calculated ΔΔG. The table is sorted by Pearson correlation in the Forward + Reverse category. The performance for PSP-GNM is shown in bold.

Method	Forward		Reverse		Forward + Reverse		Bias/Antisymmetry	
	Pearson Correlation	RMSE (Kcal/mol)	Pearson Correlation	RMSE (Kcal/mol)	Pearson Correlation	RMSE (Kcal/mol)	Bias	Antisymmetry
PremPS	0.82	1.02	0.73	1.2	0.85	1.12	−0.02	−0.93
ACDC-NN	0.63	1.44	0.6	1.5	0.71	1.47	−0.03	−0.98
ACDC-NN-Seq	0.6	1.46	0.6	1.47	0.7	1.47	0	−1
DDGun3D	0.59	1.43	0.56	1.49	0.67	1.46	−0.02	−0.99
DDGun	0.52	1.49	0.51	1.51	0.65	1.5	−0.01	−1
INPS-Seq	0.51	1.52	0.51	1.52	0.65	1.52	0	−0.99
**PSP-GNM**	**0.41**	**1.49**	**0.37**	**1.52**	**0.64**	**1.51**	**0**	**−0.97**
INPS3D	0.63	1.29	0.31	2.01	0.59	1.69	−0.51	−0.51
PoPMuSiC	0.66	1.25	0.26	2.26	0.53	1.83	−0.72	−0.29
Dynamut	0.56	1.54	0.37	1.82	0.52	1.69	−0.12	−0.59
FoldX	0.58	1.92	0.4	2.2	0.51	2.06	−0.56	−0.3
ThermoNet	0.44	1.67	0.38	1.74	0.5	1.7	−0.03	−0.9
MUpro	0.76	1.06	0.08	2.61	0.47	1.99	−0.98	−0.03
MAESTRO	0.61	1.35	0.24	2.28	0.45	1.88	−0.64	−0.33
DUET	0.63	1.28	0.18	2.38	0.45	1.91	−0.75	−0.32
mCSM	0.62	1.3	0.15	2.53	0.42	2.01	−0.92	−0.28
I-Mutant3.0-Seq	0.59	1.36	0.09	2.32	0.42	1.91	−0.65	−0.33
I-Mutant3.0	0.65	1.27	−0.05	2.43	0.39	1.94	−0.7	0.03
Dynamut2	0.63	1.29	0.05	2.48	0.39	1.98	−0.78	−0.13
SDM	0.5	1.62	0.18	2.41	0.34	2.06	−0.54	−0.44
SAAFEC-SEQ	0.72	1.14	−0.42	2.85	0.26	2.17	−0.97	0.68

**Table 9:** Performance comparisons on the Ssym+ dataset for a subset of 37 mutations with experimental temperatures ranging from 20 °C to 30 °C and pH ranging from 6.8 to 7.2. The 37 mutations had at least one contact broken, both in the wildtype and mutant forms, for the residue at the mutation position when simulating unfolding with PSP-GNM. The table is sorted by Pearson correlation in the Forward + Reverse category. The performance for PSP-GNM is shown in bold.

Method	Forward		Reverse		Forward + Reverse		Bias/Antisymmetry	
	Pearson Correlation	RMSE (Kcal/mol)	Pearson Correlation	RMSE (Kcal/mol)	Pearson Correlation	RMSE (Kcal/mol)	Bias	Antisymmetry
PremPS	0.86	0.95	0.8	1.19	0.82	1.08	0.06	−0.93
DDGun3D	0.69	1.33	0.69	1.33	0.69	1.33	0	−1
ACDC-NN	0.67	1.42	0.64	1.43	0.66	1.43	0	−0.99
INPS3D	0.49	1.58	0.64	1.53	0.59	1.56	−0.48	−0.77
DDGun	0.54	1.48	0.54	1.48	0.56	1.48	−0.01	−1
ACDC-NN-Seq	0.55	1.53	0.55	1.53	0.56	1.53	−0.01	−1
**PSP-GNM**	**0.74**	**1.48**	**0.71**	**1.55**	**0.52**	**1.51**	**0**	**−0.97**
PoPMuSiC	0.49	1.6	0.59	1.95	0.49	1.78	−0.85	−0.41
MAESTRO	0.63	1.44	0.48	2.1	0.48	1.8	−0.9	−0.44
FoldX	0.53	1.51	0.57	2.07	0.47	1.82	−0.57	−0.38
SDM	0.57	1.58	0.52	2.16	0.41	1.89	−0.53	−0.69
DUET	0.62	1.41	0.47	2.17	0.41	1.83	−0.85	−0.63
INPS-Seq	0.36	1.63	0.36	1.63	0.4	1.63	0	−1
mCSM	0.59	1.57	0.38	2.27	0.36	1.95	−1.03	−0.55
SAAFEC-SEQ	0.86	0.95	−0.45	2.33	0.34	1.78	−0.5	0.69
Dynamut2	0.66	1.43	0.22	2.15	0.34	1.82	−0.74	−0.26
Dynamut	0.45	1.59	0.34	1.81	0.32	1.7	−0.21	−0.46
I-Mutant3.0	0.17	1.81	0.22	2.14	0.17	1.98	−0.9	0.05
I-Mutant3.0-Seq	0.08	1.84	0.25	2.02	0.17	1.93	−0.79	−0.35
ThermoNet	0.35	1.67	−0.03	1.84	0.16	1.76	−0.02	−0.59
MUpro	−0.01	1.97	0.19	2.31	0.04	2.15	−1.03	0.28

**Table 10:** Datasets used in this study. The S2298 dataset was used to optimize the parameters for PSP-GNM. The remaining datasets were used to evaluate the performance of PSP-GNM.

Dataset	Description	Original Number of Mutations	Number of Proteins *	Number of Destabilizing Mutations (ΔΔG < 0)	Number of Stabilizing Mutations (ΔΔG ≥ 0)	Number of Mutations Excluded ^#^
S2298	Largest dataset and subset of the S2648 dataset that is often used for training supervised methods [53]	2298	126	1791	507	0
S350	Subset of the S2648 dataset that has been widely used for testing [53]	350	67	255	95	1
S611	Created using forward and reverse mutations from the S350 dataset [25]	611	67	331	280	2
S669	Proteins included have less than 25% sequence identity with proteins included in S2298 [45]	669	94	499	170	0
Ssym+	Forward and reverse mutation pairs observed in separate PDB IDs [13,45]	704	371	344	360	0

* Is defined as the number of unique PDB identifiers. # Mutations with non-existent wild type residues at the mutation positions in their PDB files were excluded.

## Data Availability

All the datasets used in this study and the source code for PSP-GNM are available at: https://github.com/sambitmishra0628/PSP-GNM.

## Supplementary Materials

The supporting information can be downloaded at: https://www.mdpi.com/article/10.3390/ijms231810711/s1.
