# Supplementary material for: PSP-GNM: Predicting Protein Stability Changes upon Point Mutations with a Gaussian Network Model
Source: Int J Mol Sci. 2022 Sep 14;23(18):10711. doi: 10.3390/ijms231810711 (PMC9505940; doi:10.3390/ijms231810711)
Supplement: Supplementary file 1 [file ijms-23-10711-s001.zip › ijms-1890169-supplementary.pdf]

Supporting Information

PSP-GNM: Predicting Protein Stability Changes upon Point Mutations with a Gaussian Network Model

Sambit K. Mishra[1,2]

Cancer Genomics Research Laboratory, Frederick National Laboratory, Rockville, Maryland, USA[1]

Division of Cancer Epidemiology and Genetics, National Cancer Institute, Bethesda, Maryland, USA[2]

Corresponding Author: Sambit K. Mishra (sambit.mishra@nih.gov)

Figures

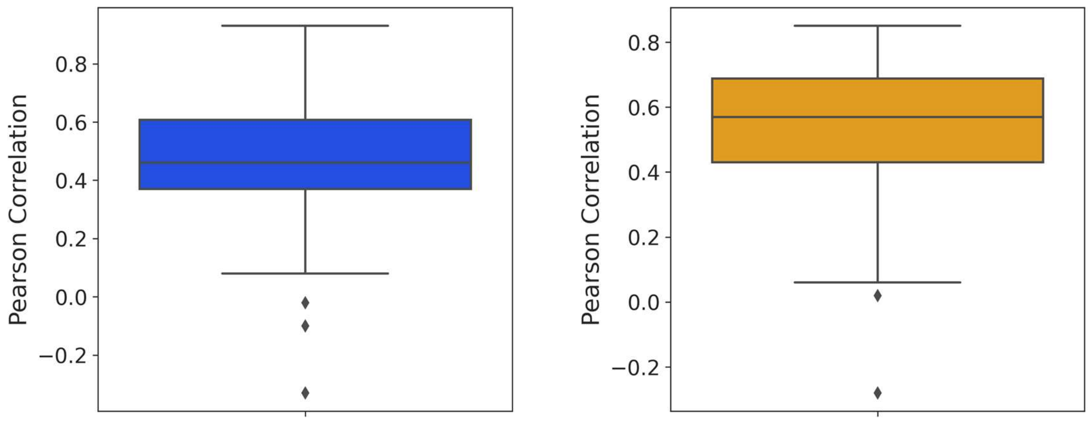

Figure S1. Extent of agreement with experimental temperature factors for GNM and PSP-GNM. Distribution of Pearson correlation between experimental B-factor and residue MSF calculated using PSP-GNM (left) and original GNM (right) is shown. The median correlation obtained for PSP-GNM is 0.46 while for GNM it is 0.57.

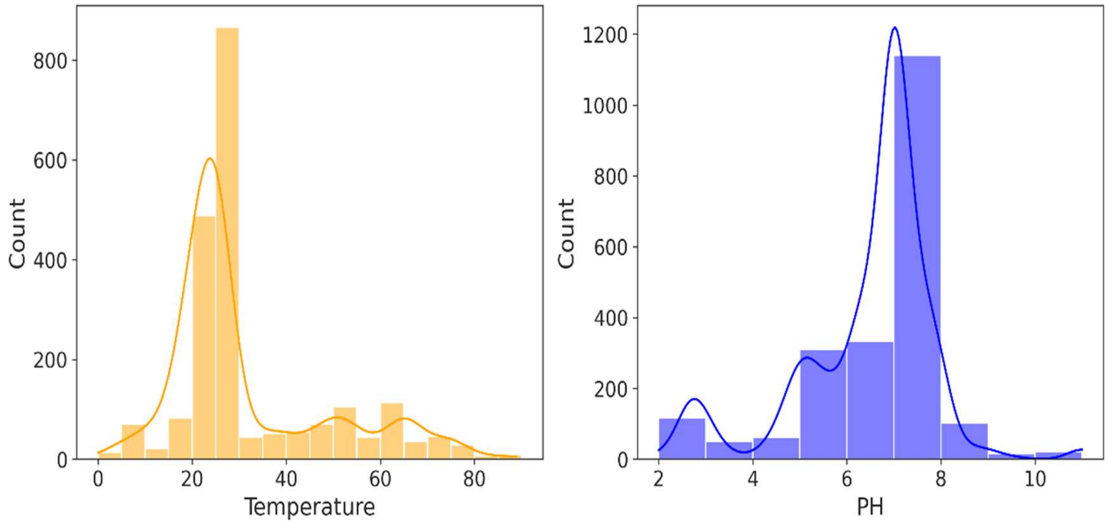

Figure S2. Distribution of experimental temperature (°C) and pH for mutants in the S2298 dataset. Mutants with a calculated ΔΔG using PSP-GNM are probed for their temperature and pH distributions. The distribution for temperature shows a peak around 25°C and the pH around 7.

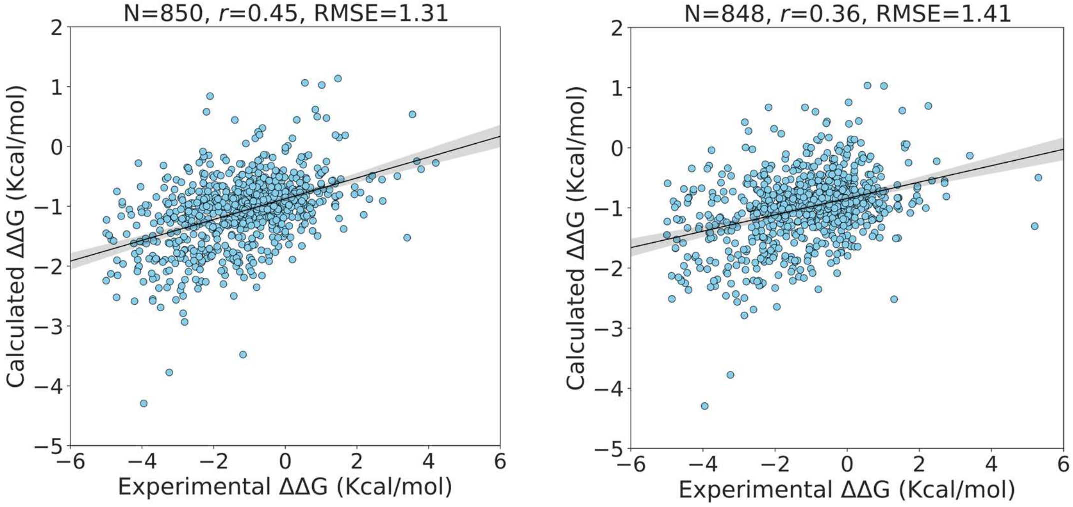

Figure S3. Agreement with experimental ΔΔG for temperature and pH ranges on the S2298 dataset. Agreement between ΔΔG from PSP-GNM and experimental measurements made at 24°C - 26°C but, varying ranges of pH (left), experimental measurements made at close to neutral pH (6.8 – 7.2) but varying ranges of temperature (right) in the S2298 dataset are shown. Only 2159 mutants with a calculated ΔΔG out of the total 2298 are considered. Units for calculated ΔΔG are in kcal/mol. The regression line of best fit and the 95% confidence interval (shaded gray) are shown.

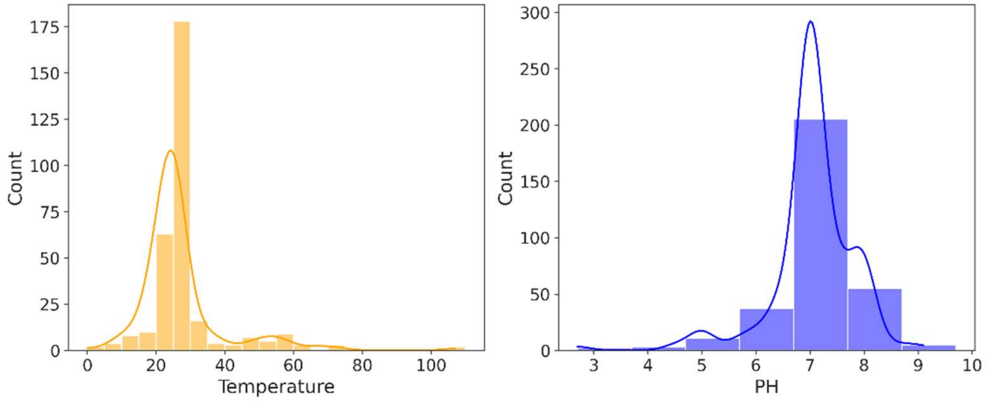

Figure S4. Distribution of experimental temperature (°C) and pH for mutants in the S350 dataset. Mutants with a PSP-GNM-calculated ΔΔG are probed for their temperature and pH distributions. The distribution for temperature shows a peak around 25°C and the pH around 7.

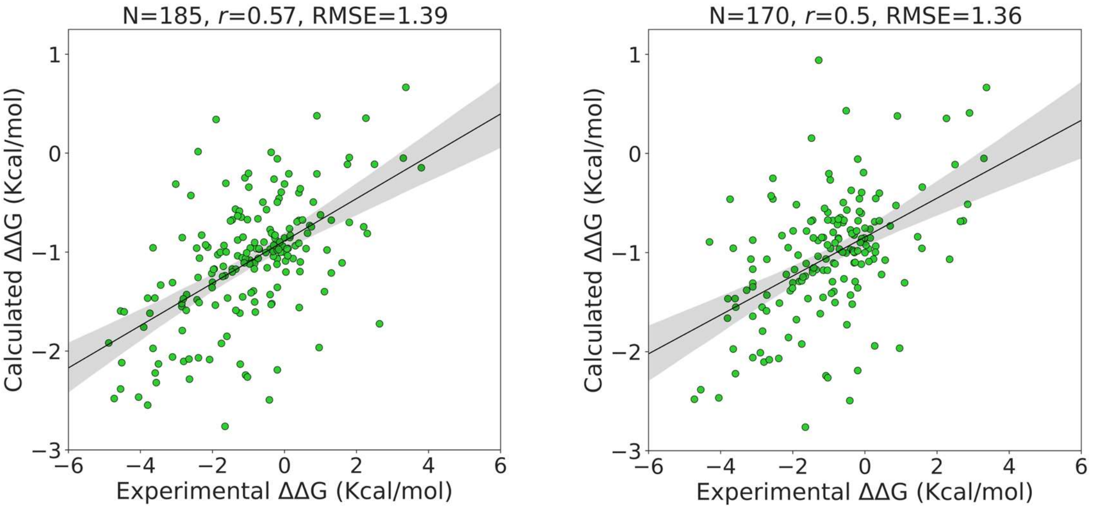

Figure S5. Agreement with experimental ΔΔG for temperature and pH ranges on the S350 dataset. The extent of agreement between ΔΔG from PSP-GNM and experimental measurements made at 24°C - 26°C but, varying ranges of pH (left), experimental measurements made at close to neutral pH (6.8 – 7.2) with varying ranges of temperature (right) for the S350 dataset. Units for calculated ΔΔG are in Kcal/mol. The regression line of best fit and the 95% confidence interval (shaded gray) are shown.

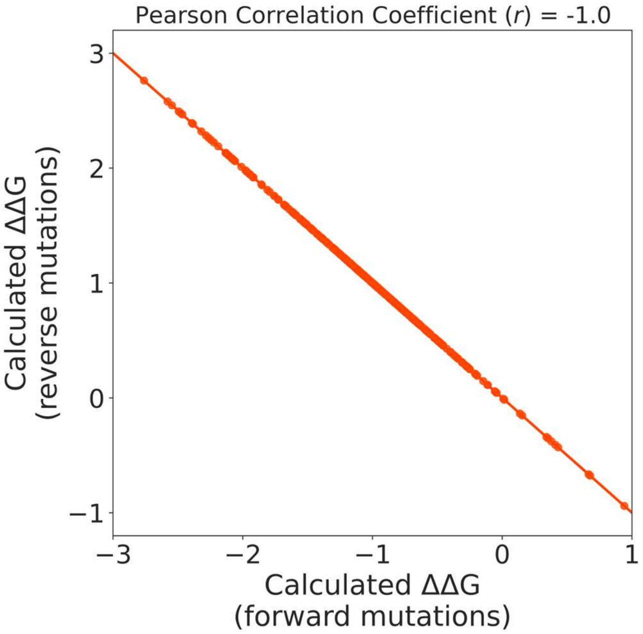

Figure S6. Anti-symmetric behavior between forward and reverse mutations. The PSP-GNM-calculated ΔΔG for the forward and reverse mutations in the S350 dataset is shown. The regression plot demonstrates anti-symmetric behavior, as one would expect, between the ΔΔG of the forward and reverse mutants. Shaded area in the plot corresponds to the 95% confidence interval. Units for the calculated ΔΔG are in kcal/mol.

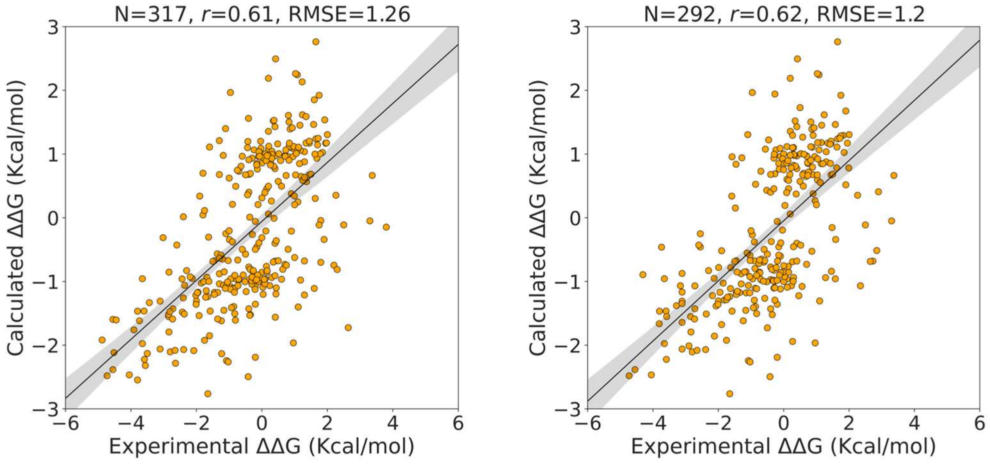

Figure S7. Impact of experimental temperature and pH on PSP-GNM for the S611 dataset. Agreement between ΔΔG from PSP-GNM and experimental measurements made at 24°C - 26°C but, varying ranges of pH (left), experimental measurements made at close to neutral pH (6.8 – 7.2) but varying ranges of temperature (right) for the S611 dataset. Units for calculated ΔΔG are in kcal/mol. The regression line of best fit and the 95% confidence interval (shaded gray) are shown.

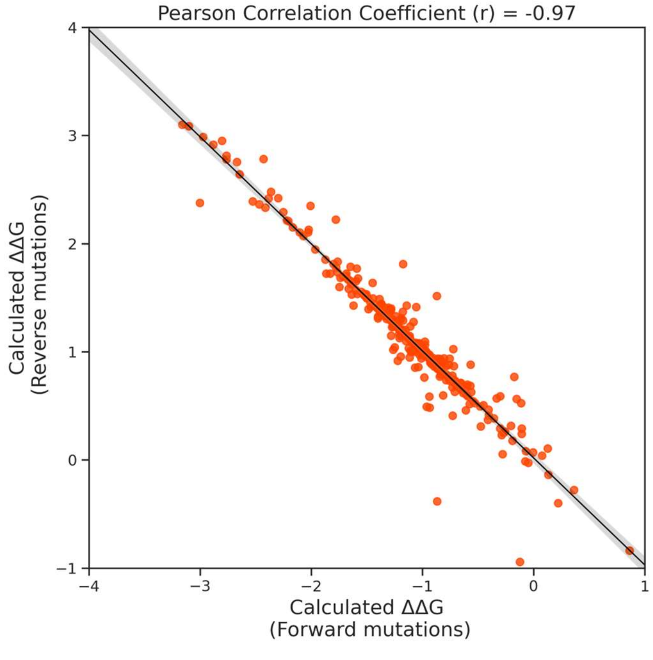

Figure S8. Anti-symmetric behavior between forward and reverse mutations on Ssym+ dataset. The PSP-GNM-calculated ΔΔG for the forward and reverse mutations in the Ssym+ dataset is shown. Only 341 mutants in each category (forward and reverse) with a PSP-GNM calculated ΔΔG are considered. The regression plot demonstrates anti-symmetric behavior, as one would expect, between the ΔΔG of the forward and reverse mutants. Shaded area in the plot corresponds to the 95% confidence interval. Units for the calculated ΔΔG are in kcal/mol.

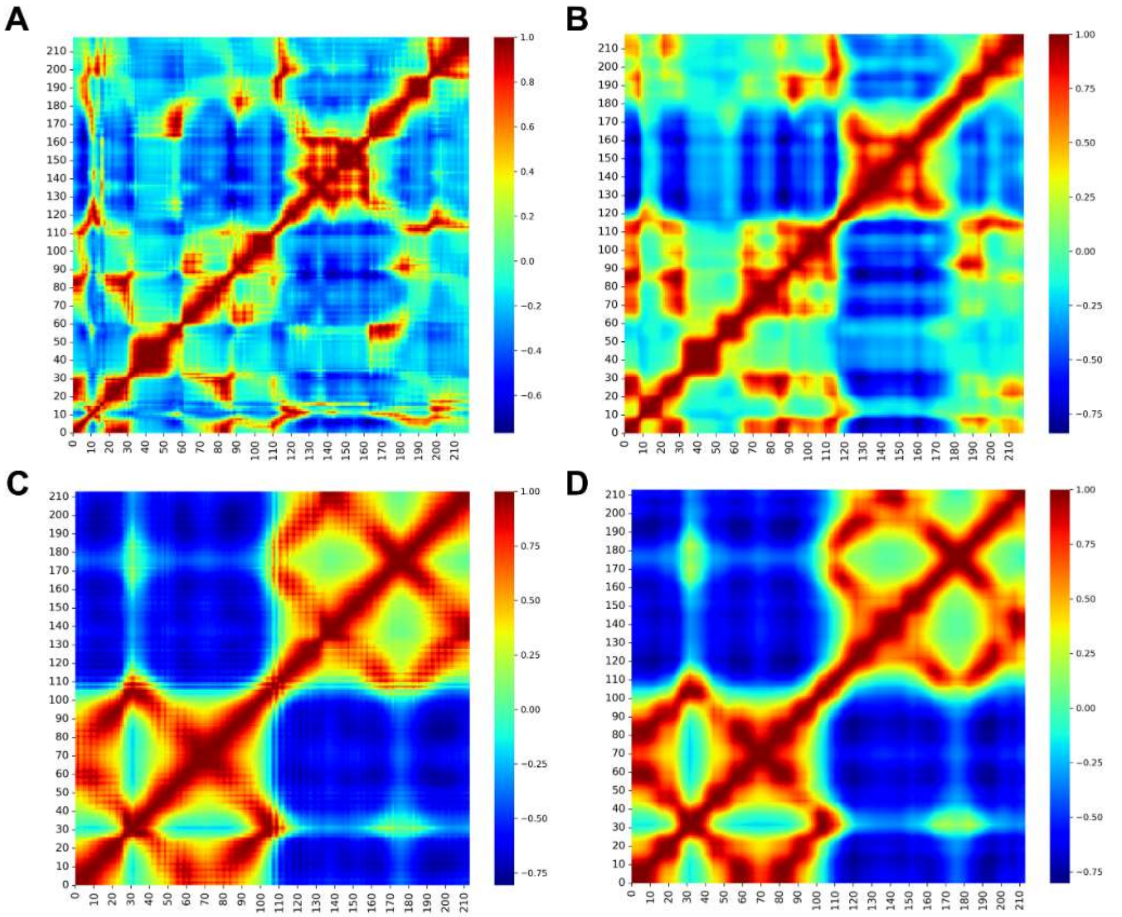

Figure S9. Comparison of correlation maps between GNM and PSP-GNM. Heatmaps of residue-residue fluctuation cross-correlations obtained for GNM (**A** and **C**) and PSP-GNM (**B** and **D**) are shown. The calculations are performed for the wildtype structures of two proteins: 1AKY (chain A) and 1CUN (chain A). The heat maps in the top row (A and B) are for 1AKY and the bottom row (C and D) are for 1CUN.

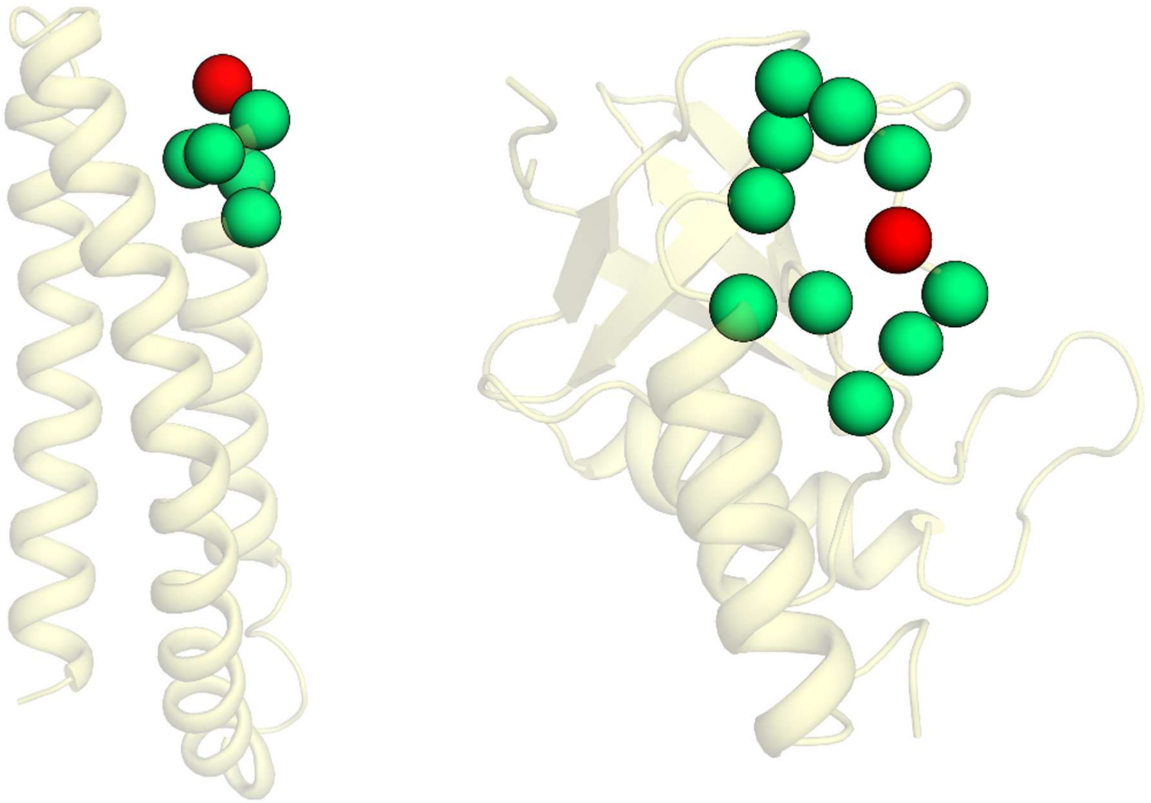

Figure S10. Cases without calculated ΔΔG. The wildtype structures of two proteins and the location of mutation positions (red) and their contacts (green) are shown for 1AJ3, chain A, residue position 10, (left) and for 1EY0, chain A, residue position 114 (right).

Tables

Table S1. Performance of PSP-GNM on the S350 dataset when using different statistical potentials.

Potential Name	AAindex Identifier	Number of Modes	Distance Cutoff (Å)	Pearson Correlation	RMSE (Kcal/mol)	Number of Predictions
Bastolla (BST)	BASU010101	10	9	0.34	1.519	215
Betancourt-Thirumalai (BT)	BETM990101	10	9	0.35	1.519	228
**Miyazawa-Jernigan (MJ)**	**MIYS960101**	**10**	**9**	**0.51**	**1.361**	**318**

Table S2. Performance comparison on the S669 dataset for all 669 mutations. Mutations without a PSP-GNM-calculated ΔΔG are assigned a theoretical value of 0.

	Forward		Reverse		Forward + Reverse		Bias/Antisymmetry	
**Method**	**Pearson Correlation**	**RMSE (Kcal/mol)**	**Pearson Correlation**	**RMSE (Kcal/mol)**	**Pearson Correlation**	**RMSE (Kcal/mol)**	**Bias**	**Antisymmetry**
ACDC-NN	0.46	1.49	0.45	1.5	0.61	1.5	-0.02	-0.98
INPS-Seq	0.43	1.52	0.43	1.53	0.61	1.52	0	-1
PremPS	0.4	1.51	0.42	1.5	0.61	1.51	0.09	-0.82
ACDC-NN-Seq	0.42	1.53	0.42	1.53	0.59	1.53	0	-1
**PSP-GNM**	**0.36**	**1.53**	**0.36**	**1.53**	**0.59**	**1.53**	**0**	**-1**
DDGun3D	0.43	1.6	0.41	1.62	0.57	1.61	-0.05	-0.96
DDGun	0.4	1.72	0.39	1.75	0.57	1.74	-0.05	-0.96
INPS3D	0.43	1.5	0.33	1.76	0.55	1.64	-0.37	-0.5
ThermoNet	0.39	1.62	0.38	1.66	0.51	1.64	-0.05	-0.85
Dynamut	0.41	1.6	0.34	1.69	0.5	1.65	-0.06	-0.58
PopMusic	0.42	1.51	0.24	2.09	0.46	1.82	-0.69	-0.31
MAESTRO	0.5	1.44	0.2	2.1	0.44	1.8	-0.57	-0.22
DUET	0.41	1.52	0.23	2.14	0.41	1.86	-0.67	-0.12
mCSM	0.36	1.54	0.22	2.3	0.37	1.96	-0.85	-0.05
I-Mutant3.0-Seq	0.34	1.56	0.21	2.23	0.36	1.92	-0.75	-0.46
Dynamut2	0.34	1.58	0.17	2.16	0.36	1.9	-0.64	0.03
MuPro	0.25	1.61	0.2	2.38	0.32	2.03	-0.95	-0.32

	Forward		Reverse		Forward + Reverse		Bias/Antisymmetry	
**Method**	**Pearson Correlation**	**RMSE (Kcal/mol)**	**Pearson Correlation**	**RMSE (Kcal/mol)**	**Pearson Correlation**	**RMSE (Kcal/mol)**	**Bias**	**Antisymmetry**
I-Mutant3.0	0.36	1.53	0.14	2.32	0.32	1.97	-0.8	-0.05
SDM	0.41	1.67	0.13	2.16	0.32	1.93	-0.4	-0.4
FoldX	0.21	2.31	0.21	2.48	0.3	2.4	-0.33	-0.2
SAAFEC-Seq	0.36	1.53	0	2.39	0.26	2.01	-0.83	-0.04

Table S3. Performance comparison on the S669 dataset for 117 mutations with a PSP-GNM calculated ΔΔG and with experimental temperatures ranging from 24°C - 26°C and pH ranging from 6.8 - 7.2.

	Forward		Reverse		Forward + Reverse		Bias/Antisymmetry	
**Method**	**Pearson Correlation**	**RMSE (Kcal/mol)**	**Pearson Correlation**	**RMSE (Kcal/mol)**	**Pearson Correlation**	**RMSE (Kcal/mol)**	**Bias**	**Antisymmetry**
PremPS	0.52	1.44	0.47	1.48	0.63	1.46	0.14	-0.82
INPS-Seq	0.45	1.53	0.45	1.53	0.61	1.53	-0.01	-1
ACDC-NN	0.43	1.52	0.41	1.54	0.58	1.53	-0.02	-0.99
ThermoNet	0.43	1.56	0.5	1.5	0.58	1.53	-0.05	-0.87
ACDC-NN-Seq	0.4	1.56	0.4	1.56	0.57	1.56	0	-1
DDGun3D	0.39	1.65	0.38	1.64	0.54	1.65	-0.03	-0.98
INPS3D	0.53	1.45	0.24	1.84	0.54	1.65	-0.47	-0.5
PopMusic	0.54	1.47	0.2	2.13	0.53	1.83	-0.9	-0.49
Dynamut	0.43	1.55	0.3	1.67	0.51	1.61	-0.02	-0.58
DDGun	0.32	1.83	0.33	1.87	0.5	1.85	-0.05	-0.95
**PSP-GNM**	**0.23**	**1.64**	**0.23**	**1.64**	**0.5**	**1.64**	**0**	**-1**
DUET	0.6	1.37	0.13	2.23	0.48	1.85	-0.85	-0.25
MAESTRO	0.55	1.44	0.11	2.2	0.46	1.86	-0.8	-0.25
mCSM	0.58	1.4	0.16	2.37	0.46	1.95	-1.01	-0.23
FoldX	0.44	2.15	0.18	2.21	0.45	2.18	-0.41	-0.37
SAAFEC-Seq	0.46	1.51	0.12	2.31	0.43	1.95	-0.96	-0.29
Dynamut2	0.34	1.58	0.17	2.16	0.36	1.9	-0.64	0.03
I-Mutant3.0-Seq	0.34	1.61	0.04	2.27	0.34	1.96	-0.86	-0.37
SDM	0.47	1.59	-0.07	2.28	0.32	1.97	-0.54	-0.36
I-Mutant3.0	0.46	1.51	-0.09	2.49	0.31	2.06	-1.03	-0.17
MuPro	0.12	1.75	-0.01	2.38	0.2	2.09	-0.98	-0.37

Table S4. Performance comparison on the Ssym+ dataset for all the 352 mutations.

	Forward		Reverse		Forward + Reverse		Bias/Antisymmetry	
**Method**	**Pearson Correlation**	**RMSE (Kcal/mol)**	**Pearson Correlation**	**RMSE (Kcal/mol)**	**Pearson Correlation**	**RMSE (Kcal/mol)**	**Bias**	**Antisymmetry**
PremPS	0.81	1.05	0.73	1.21	0.84	1.14	-0.02	-0.93
ACDC-NN	0.62	1.46	0.6	1.51	0.7	1.48	-0.03	-0.98
ACDC-NN-Seq	0.6	1.47	0.6	1.47	0.69	1.47	0	-1
DDGun3D	0.58	1.45	0.56	1.49	0.66	1.47	-0.02	-0.99
DDGun	0.51	1.51	0.51	1.51	0.64	1.51	-0.01	-1
INPS-Seq	0.5	1.53	0.51	1.52	0.64	1.53	0	-0.99
**PSP-GNM**	**0.41**	**1.5**	**0.36**	**1.53**	**0.63**	**1.52**	**0**	**-0.96**
INPS3D	0.62	1.31	0.32	2.01	0.58	1.69	-0.51	-0.52
PoPMuSiC	0.65	1.27	0.28	2.25	0.53	1.83	-0.72	-0.3
FoldX	0.57	1.94	0.41	2.2	0.51	2.07	-0.58	-0.26
Dynamut	0.55	1.56	0.38	1.82	0.51	1.7	-0.12	-0.57
ThermoNet	0.45	1.67	0.38	1.74	0.5	1.7	-0.02	-0.89
MUpro	0.76	1.06	0.1	2.59	0.47	1.98	-0.96	-0.05
DUET	0.63	1.29	0.2	2.37	0.45	1.91	-0.74	-0.3
MAESTRO	0.6	1.37	0.25	2.27	0.44	1.87	-0.63	-0.32
I-Mutant3.0-Seq	0.59	1.37	0.11	2.31	0.42	1.9	-0.65	-0.34
mCSM	0.61	1.31	0.16	2.52	0.41	2.01	-0.91	-0.27
I-Mutant3.0	0.64	1.29	-0.03	2.41	0.39	1.93	-0.69	0.01
Dynamut2	0.62	1.3	0.07	2.48	0.39	1.98	-0.78	-0.12
SDM	0.51	1.62	0.2	2.39	0.35	2.04	-0.54	-0.44
SAAFEC-SEQ	0.71	1.16	-0.39	2.83	0.26	2.17	-0.97	0.66

Table S5. Extent of similarity between the S350 and S2298 datasets. For each protein in the S350 dataset, the protein with best sequence match in the S2298 dataset is reported. The best match was obtained by using the --subject_besthit argument in BLASTp.

Query* ID	Subject* ID	% Identity	Alignment Length	Query start	Query end	Subject start	Subject end	E value	Bit score	% Coverage
1AJ3A	1AJ3A	100	98	1	98	1	98	4.68E-71	199	100
1AKYA	1AKYA	100	218	1	218	1	218	7.32E-166	448	100
1AONU	1AONU	100	97	1	97	1	97	6.76E-67	188	100
1APSA	1APSA	100	98	1	98	1	98	7.15E-72	201	100
1BNIA	1BNIA	100	108	1	108	1	108	1.87E-80	223	100
1BTAA	1BTAA	100	89	1	89	1	89	3.85E-65	183	100
1BVCA	1BVCA	100	153	1	153	1	153	8.15E-112	306	100
1C9OA	1C9OA	100	66	1	66	1	66	1.11E-45	132	100
1CEYA	1CEYA	100	128	1	128	1	128	7.53E-93	256	100
1CSEI	1CSEI	100	63	1	63	1	63	1.75E-44	129	100
1CSPA	1CSPA	100	67	1	67	1	67	5.52E-46	133	100
1CUNA	1CUNA	100	213	1	213	1	213	9.82E-160	432	100
1DKTA	1DKTA	100	72	1	72	1	72	1.13E-52	150	100
1E65A	1E65A	100	128	1	128	1	128	2.06E-97	268	100
1EY0A	1EY0A	100	136	1	136	1	136	8.64E-102	279	100
1FNAA	1FNAA	100	91	1	91	1	91	9.41E-64	180	100
1FTGA	1FTGA	100	168	1	168	1	168	1.45E-123	337	100
1G4IA	1G4IA	100	123	1	123	1	123	6.15E-91	251	100
1H7MA	1H7MA	100	99	1	99	1	99	4.56E-71	199	100
1HFZA	1HFZA	100	123	1	123	1	123	6.06E-92	253	100
1HMEA	1HMEA	100	77	1	77	1	77	3.97E-55	157	100
1HMKA	1HMKA	100	121	1	121	1	121	2.38E-90	249	100
1HMSA	1HMSA	100	131	1	131	1	131	1.01E-95	263	100
1IETA					No significant hits					
1IFCA	1IFCA	100	131	1	131	1	131	2.46E-95	263	100
1IGVA	1IGVA	100	75	1	75	1	75	9.97E-50	143	100
1IHBA	1IHBA	100	156	1	156	1	156	3.78E-115	315	100
1IMQA	1IMQA	100	86	1	86	1	86	3.07E-64	181	100
1IROA					No significant hits					
1JIWI					No significant hits					
1K9QA	1K9QA	100	40	1	40	1	40	1.43E-28	87.4	100
1KFWA					No significant hits					
1LNIA	1LNIA	100	96	1	96	1	96	1.88E-70	197	100

Query* ID	Subject* ID	% Identity	Alignment Length	Query start	Query end	Subject start	Subject end	E value	Bit score	% Coverage
1LZ1A	1LZ1A	100	130	1	130	1	130	1.65E-97	268	100
1MGRA	1MGRA	100	97	1	97	1	97	6.85E-73	203	100
1MJCA	1MJCA	100	69	1	69	1	69	2.08E-48	139	100
1MSIA	1MSIA	100	66	1	66	1	66	1.32E-44	129	100
1OIAA	1OIAA	100	90	1	90	1	90	1.85E-66	186	100
1P2PA	1P2PA	100	124	1	124	1	124	4.74E-93	256	100
1QLPA	1QLPA	100	372	1	372	1	372	0	759	100
1RG8A	1RG8A	100	141	1	141	1	141	6.14E-108	295	100
1RISA	1RISA	100	97	1	97	1	97	6.57E-69	193	100
1RN1C	1RN1C	100	104	1	104	1	104	2.58E-76	213	100
1RTBA	1RTBA	100	124	1	124	1	124	9.97E-94	258	100
1RTP1					No significant hits					
1SHFA	1SHFA	100	59	1	59	1	59	1.65E-42	124	100
1TITA	1TITA	100	89	1	89	1	89	1.00E-64	182	100
1TTQA	1TTQA	100	256	1	256	1	256	0	518	100
1UZCA	1UZCA	100	69	1	69	1	69	8.38E-48	138	100
1YYJA	1YYJA	100	106	1	106	1	106	2.29E-76	213	100
1ZG4A	1ZG4A	100	263	1	263	1	263	0	541	100
1ZNJB	1ZNJB	100	30	1	30	1	30	3.84E-20	65.5	100
2A36A	2A36A	100	59	1	59	1	59	2.51E-42	123	100
2DRIA	2DRIA	100	271	1	271	1	271	0	537	100
2IMMA	2IMMA	100	114	1	114	1	114	2.46E-85	236	100
2LZMA	2LZMA	100	164	1	164	1	164	5.22E-125	340	100
2NVHA	2NVHA	100	152	1	152	1	152	4.84E-115	314	100
2RN2A	2RN2A	100	155	1	155	1	155	1.12E-119	326	100
2TRXA	2TRXA	100	108	1	108	1	108	7.23E-79	219	100
3GLYA	3GLYA	100	470	1	470	1	470	0	950	100
3MBPA	3MBPA	100	370	1	370	1	370	0	748	100
3PGKA	3PGKA	100	415	1	415	1	415	0	832	100
3SILA	3SILA	100	379	1	379	1	379	0	783	100
4LYZA	4LYZA	100	129	1	129	1	129	4.12E-96	265	100
5DFRA	5DFRA	100	154	1	154	1	154	1.87E-117	320	100
5PTIA	5PTIA	100	58	1	58	1	58	1.75E-41	121	100

*Query ID: PDB ID + Chain ID for proteins in the S350 dataset
*Subject ID: PDB ID + Chain ID for best hit in the S2298 dataset

Table S6. Extent of similarity between the S669 and S2298 datasets. For each protein in the S669 dataset, the protein with the best sequence match in the S2298 dataset is reported. The best match was obtained by using the --subject_besthit argument in BLASTp.

Query* ID	Subject* ID	% Identity	Alignment Length	Query start	Query end	Subject start	Subject end	E value	Bit score	% Coverage
1A0FA					No significant hits					
1A7VA					No significant hits					
1BA3A					No significant hits					
1BFMA					No significant hits					
1BNLA					No significant hits					
1D5GA					No significant hits					
1DIVA					No significant hits					
1DXXA					No significant hits					
1EKGA					No significant hits					
1F8IA					No significant hits					
1FH5H	2IMMA	31.034	87	14	93	21	97	0.0001 73	31.6	43.9
1FRDA					No significant hits					
1FT8A					No significant hits					
1FXAA					No significant hits					
1G3PA					No significant hits					
1GLUA					No significant hits					
1GUAB					No significant hits					
1GWYA					No significant hits					
1H0XA					No significant hits					
1HCQA					No significant hits					
1IOJA					No significant hits					
1IR3A					No significant hits					
1ITMA					No significant hits					
1IV7B					No significant hits					
1IV9A					No significant hits					
1J8IA					No significant hits					
1JL9A					No significant hits					
1JLVA					No significant hits					
1L6HA					No significant hits					
1LVMA					No significant hits					
1N18A					No significant hits					
1N88A					No significant hits					
1NM1A					No significant hits					
1O1UA	1HMSA	30.769	91	1	91	3	92	2.81E-09	43.9	71.7
1O6XA					No significant hits					
1OSIA					No significant hits					
1PFLA					No significant hits					
1PREA					No significant hits					
1PRGA					No significant hits					
1R2YA					No significant hits					
1R6RA					No significant hits					
1SPDA					No significant hits					
1X0JA					No significant hits					
1XWSA					No significant hits					
1XXNA					No significant hits					
1XZOA					No significant hits					
2ARFA					No significant hits					

Query* ID	Subject* ID	% Identity	Alignment Length	Query start	Query end	Subject start	Subject end	E value	Bit score	% Coverage
2BJDA	1APSA	31.579	76	2	76	6	81	1.16E-11	48.1	84.4
2C9QA					No significant hits					
2CLRB					No significant hits					
2DVVA					No significant hits					
2H3FA					No significant hits					
2HBBA					No significant hits					
2JIEA					No significant hits					
2JUCA					No significant hits					
2KJ3A					No significant hits					
2KS4A					No significant hits					
2LTBA					No significant hits					
2M5SA					No significant hits					
2MPCA					No significant hits					
2N7ZA					No significant hits					
2NTEA					No significant hits					
2OUOA					No significant hits					
2PR5A					No significant hits					
2PTLA					No significant hits					
2RPNA	2A36A	43.103	58	4	59	3	57	2.49E-11	45.4	98.3
2VY0A					No significant hits					
2WQGA					No significant hits					
2ZTAA					No significant hits					
3BCIA					No significant hits					
3BN0A					No significant hits					
3C2IA					No significant hits					
3D2AA					No significant hits					
3D3BA					No significant hits					
3DV0I					No significant hits					
3ECUA					No significant hits					
3FISA					No significant hits					
3G1GA					No significant hits					
3K82A					No significant hits					
3L15B					No significant hits					
3MONB					No significant hits					
3O39A					No significant hits					
3S4MA					No significant hits					
3S92A					No significant hits					
4BJXA					No significant hits					
4BUQA					No significant hits					
4HE7A					No significant hits					
4N6V0					No significant hits					
4WAAA					No significant hits					
4YEEA					No significant hits					
4YEFA					No significant hits					
5JXBA					No significant hits					
5OAQA					No significant hits					
5VP3A					No significant hits					

*Query ID: PDB ID + Chain ID for proteins in the S669 dataset
*Subject ID: PDB ID + Chain ID for best hit in the S2298 dataset

Table S7. Extent of similarity between the Ssym+ forward and S2298 datasets. For each protein in the Ssym+ forward dataset, the protein with the best sequence match in the S2298 dataset is reported. The best match was obtained by using the --subject_besthit argument in BLASTp.

Query* ID	Subject* ID	% Identity	Alignment Length	Query start	Query end	Subject start	Subject end	E value	Bit score	% Coverage
1amqA	1AMQA	100	396	1	396	1	396	0	824	100
1arrA	1ARRA	100	53	1	53	1	53	7.46E-37	109	100
1bniA	1BNIA	100	108	1	108	1	108	1.87E-80	223	100
1ceyA	1CEYA	100	128	1	128	1	128	7.53E-93	256	100
1ey0A	1EY0A	100	136	1	136	1	136	8.64E-102	279	100
1ihbA	1IHBA	100	156	1	156	1	156	3.78E-115	315	100
1iobA	2NVHA	100	152	1	152	1	152	3.75E-115	314	99.3
1l63A	2LZMA	98.765	162	1	162	1	162	3.30E-121	330	100
1lz1A	1LZ1A	100	130	1	130	1	130	1.65E-97	268	100
1n0jA	1N0JA	100	198	1	198	1	198	3.96E-152	412	100
1oh0A	1OH0A	100	125	1	125	1	125	1.35E-94	260	100
1rn1C	1RN1C	100	104	1	104	1	104	2.58E-76	213	100
1vqbA	1VQBA	100	86	1	86	1	86	3.93E-63	177	100
2lzmA	2LZMA	100	164	1	164	1	164	5.22E-125	340	100
2rn2A	2RN2A	100	155	1	155	1	155	1.12E-119	326	100
3zcfA					No significant hits					
4bvmA	1HMSA	63.566	129	4	132	2	130	1.27E-61	177	97
4lyzA	4LYZA	100	129	1	129	1	129	4.12E-96	265	100
5ptiA	5PTIA	100	58	1	58	1	58	1.75E-41	121	100

*Query ID: PDB ID + Chain ID for proteins in the Ssym+ forward dataset
*Subject ID: PDB ID + Chain ID for best hit in the S2298 dataset

Table S8. Extent of similarity between the Ssym+ reverse and S2298 datasets. For each protein in the Ssym+ forward dataset, the protein with the best sequence match in the S2298 dataset is reported. The best match was obtained by using the --subject_besthit argument in BLASTp.

Query* ID	Subject * ID	% Identity	Alignment Length	Query start	Query end	Subject start	Subject end	E value	Bit score	% Coverage
107lA	2LZMA	98.148	162	1	162	1	162	2.00E-120	328	100
108lA	2LZMA	98.148	162	1	162	1	162	3.65E-120	328	100
109lA	2LZMA	98.148	162	1	162	1	162	1.64E-120	329	100
110lA	2LZMA	98.148	162	1	162	1	162	4.08E-120	328	100
111lA	2LZMA	98.148	162	1	162	1	162	1.57E-120	329	100

Query* ID	Subject * ID	% Identity	Alignment Length	Query start	Query end	Subject start	Subject end	E value	Bit score	% Coverage
112lA	2LZMA	98.148	162	1	162	1	162	2.23E-120	328	100
113lA	2LZMA	98.148	162	1	162	1	162	2.35E-120	328	100
114lA	2LZMA	98.148	162	1	162	1	162	7.04E-121	330	100
115lA	2LZMA	98.148	162	1	162	1	162	2.06E-120	328	100
118lA	2LZMA	98.148	162	1	162	1	162	7.20E-121	330	100
119lA	2LZMA	98.148	162	1	162	1	162	7.20E-121	330	100
120lA	2LZMA	98.148	162	1	162	1	162	7.20E-121	330	100
122lA	2LZMA	98.148	162	1	162	1	162	7.20E-121	330	100
123lA	2LZMA	98.148	162	1	162	1	162	7.20E-121	330	100
125lA	2LZMA	98.148	162	1	162	1	162	7.20E-121	330	100
126lA	2LZMA	98.148	162	1	162	1	162	1.36E-120	329	100
127lA	2LZMA	98.148	162	1	162	1	162	1.36E-120	329	100
128lA	2LZMA	98.148	162	1	162	1	162	1.36E-120	329	100
137lA	2LZMA	98.148	162	1	162	1	162	3.99E-120	328	100
149lA	2LZMA	99.39	164	1	164	1	164	1.45E-124	339	100
150lA	2LZMA	99.39	164	1	164	1	164	2.56E-124	338	100
172lA	2LZMA	99.39	164	1	164	1	164	2.99E-124	338	100
1b7sA	1LZ1A	99.231	130	1	130	1	130	9.05E-97	266	100
1banA	1BNIA	99.074	108	1	108	1	108	4.44E-80	222	100
1baoA	1BNIA	99.074	108	1	108	1	108	4.59E-80	222	100
1bnsA	1BNIA	99.074	108	1	108	1	108	8.40E-80	221	100
1bptA	5PTIA	98.214	56	1	56	1	56	4.27E-39	115	100
1brgA	1BNIA	99.074	108	1	108	1	108	9.69E-80	221	100
1brhA	1BNIA	99.074	108	1	108	1	108	1.11E-79	221	100
1briA	1BNIA	99.074	108	1	108	1	108	1.12E-79	221	100
1brjA	1BNIA	99.074	108	1	108	1	108	1.12E-79	221	100
1brkA	1BNIA	99.074	108	1	108	1	108	1.12E-79	221	100
1bsaA	1BNIA	99.065	107	1	107	2	108	1.25E-79	221	100
1bsbA	1BNIA	99.074	108	1	108	1	108	2.80E-80	223	100
1bscA	1BNIA	99.074	108	1	108	1	108	2.80E-80	223	100
1bsdA	1BNIA	99.065	107	1	107	2	108	1.25E-79	221	100
1bseA	1BNIA	99.074	108	1	108	1	108	5.42E-80	222	100
1btiA	5PTIA	98.276	58	1	58	1	58	1.88E-40	118	100
1ctwA	2LZMA	98.148	162	1	162	1	162	1.41E-120	329	100

Query* ID	Subject * ID	% Identity	Alignment Length	Query start	Query end	Subject start	Subject end	E value	Bit score	% Coverage
1cu0A	2LZMA	98.148	162	1	162	1	162	8.21E-121	330	100
1cu2A	2LZMA	98.148	162	1	162	1	162	5.72E-121	330	100
1cu3A	2LZMA	98.148	162	1	162	1	162	1.03E-120	329	100
1cu5A	2LZMA	98.148	162	1	162	1	162	5.72E-121	330	100
1cu6A	2LZMA	98.148	162	1	162	1	162	1.52E-120	329	100
1cupA	2LZMA	98.148	162	1	162	1	162	8.21E-121	330	100
1cuqA	2LZMA	98.148	162	1	162	1	162	1.03E-120	329	100
1cv0A	2LZMA	98.148	162	1	162	1	162	1.83E-120	329	100
1cv1A	2LZMA	98.148	162	1	162	1	162	1.03E-120	329	100
1cv3A	2LZMA	98.148	162	1	162	1	162	5.72E-121	330	100
1cv4A	2LZMA	98.148	162	1	162	1	162	5.72E-121	330	100
1cv5A	2LZMA	98.148	162	1	162	1	162	5.72E-121	330	100
1cv6A	2LZMA	98.148	162	1	162	1	162	1.03E-120	329	100
1cvkA	2LZMA	98.148	162	1	162	1	162	1.52E-120	329	100
1d2wA	2LZMA	98.148	162	1	162	1	162	8.21E-121	330	100
1d2yA	2LZMA	98.148	162	1	162	1	162	8.21E-121	330	100
1d3jA	2LZMA	98.148	162	1	162	1	162	5.72E-121	330	100
1dmmA	1OH0A	96.8	125	2	123	1	125	1.02E-89	248	101.6
1dmqA	1OH0A	96.8	125	2	123	1	125	1.02E-89	248	101.6
1dyaA	2LZMA	99.383	162	1	162	1	162	2.24E-122	333	100
1dybA	2LZMA	99.383	162	1	162	1	162	1.92E-122	334	100
1dycA	2LZMA	99.383	162	1	162	1	162	2.31E-123	336	100
1dydA	2LZMA	99.383	162	1	162	1	162	4.57E-123	335	100
1dyeA	2LZMA	99.383	162	1	162	1	162	7.74E-123	335	100
1dyfA	2LZMA	99.383	162	1	162	1	162	4.28E-123	335	100
1dygA	2LZMA	99.383	162	1	162	1	162	1.24E-122	334	100
1e6kA	1CEYA	98.438	128	3	130	1	128	1.64E-91	253	98.5
1e6lA	1CEYA	99.213	127	1	127	2	128	2.95E-91	252	100
1e6mA	1CEYA	98.438	128	1	128	1	128	1.90E-91	253	100
1ey4A	1EY0A	99.265	136	1	136	1	136	3.48E-101	278	100
1ey5A	1EY0A	99.259	135	1	135	2	136	1.87E-100	276	100
1ey6A	1EY0A	99.259	135	1	135	2	136	2.78E-100	275	100
1ey7A	1EY0A	99.259	135	1	135	2	136	1.19E-100	276	100
1fanA	5PTIA	98.276	58	1	58	1	58	1.88E-40	118	100

Query* ID	Subject * ID	% Identity	Alignment Length	Query start	Query end	Subject start	Subject end	E value	Bit score	% Coverage
1flqA	4LYZA	99.225	129	1	129	1	129	2.36E-95	263	100
1fluA	4LYZA	99.225	129	1	129	1	129	2.36E-95	263	100
1flwA	4LYZA	99.225	129	1	129	1	129	2.36E-95	263	100
1flyA	4LYZA	99.225	129	1	129	1	129	2.36E-95	263	100
1fn5A	4LYZA	99.225	129	1	129	1	129	2.36E-95	263	100
1g06A	2LZMA	98.171	164	1	164	1	164	5.31E-122	333	100
1g07A	2LZMA	98.148	162	1	162	1	162	4.03E-120	328	100
1g0gA	2LZMA	98.148	162	1	162	1	162	1.42E-120	329	100
1g0jA	2LZMA	98.171	164	1	164	1	164	2.63E-122	333	100
1g0kA	2LZMA	98.171	164	1	164	1	164	1.78E-121	331	100
1g0lA	2LZMA	98.171	164	1	164	1	164	4.71E-122	333	100
1g0mA	2LZMA	98.148	162	1	162	1	162	2.00E-120	328	100
1g0pA	2LZMA	98.171	164	1	164	1	164	1.76E-121	331	100
1g0qA	2LZMA	98.171	164	1	164	1	164	1.55E-122	334	100
1g1vA	2LZMA	98.148	162	1	162	1	162	1.36E-120	329	100
1g1wA	2LZMA	98.148	162	1	162	1	162	2.35E-120	328	100
1gayA	1LZ1A	99.231	130	1	130	1	130	1.97E-96	265	100
1gazA	1LZ1A	99.231	130	1	130	1	130	2.27E-97	268	100
1gb0A	1LZ1A	99.231	130	1	130	1	130	4.20E-97	267	100
1gb2A	1LZ1A	99.231	130	1	130	1	130	3.84E-97	267	100
1gb3A	1LZ1A	99.231	130	1	130	1	130	6.88E-97	266	100
1gb5A	1LZ1A	99.231	130	1	130	1	130	1.97E-96	265	100
1gb6A	1LZ1A	99.231	130	1	130	1	130	2.27E-97	268	100
1gb7A	1LZ1A	99.231	130	1	130	1	130	4.20E-97	267	100
1gb8A	1LZ1A	99.231	130	1	130	1	130	3.84E-97	267	100
1gb9A	1LZ1A	99.231	130	1	130	1	130	6.88E-97	266	100
1gboA	1LZ1A	99.231	130	1	130	1	130	1.97E-96	265	100
1gbwA	1LZ1A	99.231	130	1	130	1	130	2.27E-97	268	100
1gbxA	1LZ1A	99.231	130	1	130	1	130	4.20E-97	267	100
1gbyA	1LZ1A	99.231	130	1	130	1	130	3.84E-97	267	100
1gbzA	1LZ1A	99.231	130	1	130	1	130	6.88E-97	266	100
1gf8A	1LZ1A	99.231	130	1	130	1	130	9.05E-97	266	100
1gf9A	1LZ1A	99.231	130	1	130	1	130	1.10E-96	266	100
1gfaA	1LZ1A	99.231	130	1	130	1	130	2.04E-96	265	100

Query* ID	Subject * ID	% Identity	Alignment Length	Query start	Query end	Subject start	Subject end	E value	Bit score	% Coverage
1gfeA	1LZ1A	99.231	130	1	130	1	130	1.87E-96	265	100
1gfgA	1LZ1A	99.231	130	1	130	1	130	1.99E-96	265	100
1gfhA	1LZ1A	99.231	130	1	130	1	130	1.10E-96	266	100
1gfjA	1LZ1A	99.231	130	1	130	1	130	2.04E-96	265	100
1gfkA	1LZ1A	99.231	130	1	130	1	130	1.87E-96	265	100
1gfrA	1LZ1A	99.231	130	1	130	1	130	1.99E-96	265	100
1gftA	1LZ1A	99.231	130	1	130	1	130	1.10E-96	266	100
1gfuA	1LZ1A	99.231	130	1	130	1	130	2.04E-96	265	100
1gfvA	1LZ1A	99.231	130	1	130	1	130	1.87E-96	265	100
1gobA	2RN2A	99.355	155	1	155	1	155	5.45E-119	324	100
1hemA	4LYZA	99.225	129	1	129	1	129	1.26E-95	263	100
1heoA	4LYZA	99.225	129	1	129	1	129	6.98E-96	264	100
1herA	4LYZA	99.225	129	1	129	1	129	1.52E-95	263	100
1hibA	2NVHA	98.667	150	1	149	3	152	1.24E-109	300	100
1hnlA	1LZ1A	99.231	130	1	130	1	130	1.42E-96	266	100
1i6sA	2LZMA	98.148	162	1	162	1	162	3.03E-120	328	100
1inuA	1LZ1A	99.231	130	1	130	1	130	1.99E-96	265	100
1iosA	4LYZA	99.225	129	1	129	1	129	3.55E-95	262	100
1iotA	4LYZA	99.225	129	1	129	1	129	1.49E-95	263	100
1ir7A	4LYZA	99.225	129	1	129	1	129	1.14E-95	263	100
1ir8A	4LYZA	99.225	129	1	129	1	129	1.14E-95	263	100
1ir9A	4LYZA	99.225	129	1	129	1	129	1.14E-95	263	100
1kabA	1EY0A	99.265	136	1	136	1	136	9.36E-101	277	100
1kvaA	2RN2A	99.355	155	1	155	1	155	1.31E-118	323	100
1kvbA	2RN2A	99.355	155	1	155	1	155	1.86E-118	323	100
1kvcA	2RN2A	99.355	155	1	155	1	155	5.88E-119	324	100
1l00A	2LZMA	99.39	164	1	164	1	164	5.83E-124	338	100
1l02A	2LZMA	99.39	164	1	164	1	164	2.65E-124	338	100
1l03A	2LZMA	99.39	164	1	164	1	164	3.72E-124	338	100
1l04A	2LZMA	99.39	164	1	164	1	164	4.95E-124	338	100
1l06A	2LZMA	99.39	164	1	164	1	164	4.02E-124	338	100
1l07A	2LZMA	99.39	164	1	164	1	164	6.73E-124	337	100
1l08A	2LZMA	99.39	164	1	164	1	164	6.44E-124	337	100
1l09A	2LZMA	99.39	164	1	164	1	164	3.56E-124	338	100

Query* ID	Subject * ID	% Identity	Alignment Length	Query start	Query end	Subject start	Subject end	E value	Bit score	% Coverage
1l10A	2LZMA	99.39	164	1	164	1	164	2.83E-124	338	100
1l11A	2LZMA	99.39	164	1	164	1	164	3.68E-124	338	100
1l12A	2LZMA	99.39	164	1	164	1	164	3.26E-124	338	100
1l13A	2LZMA	99.39	164	1	164	1	164	6.80E-124	337	100
1l14A	2LZMA	99.39	164	1	164	1	164	9.86E-125	340	100
1l15A	2LZMA	99.39	164	1	164	1	164	2.56E-124	338	100
1l16A	2LZMA	99.39	164	1	164	1	164	6.16E-124	338	100
1l17A	2LZMA	99.39	164	1	164	1	164	6.71E-125	340	100
1l18A	2LZMA	99.39	164	1	164	1	164	4.02E-124	338	100
1l19A	2LZMA	99.39	164	1	164	1	164	3.26E-124	338	100
1l20A	2LZMA	99.39	164	1	164	1	164	2.71E-124	338	100
1l21A	2LZMA	99.39	164	1	164	1	164	4.43E-124	338	100
1l22A	2LZMA	99.39	164	1	164	1	164	6.23E-124	338	100
1l23A	2LZMA	99.39	164	1	164	1	164	3.05E-124	338	100
1l24A	2LZMA	99.39	164	1	164	1	164	3.19E-124	338	100
1l33A	2LZMA	99.39	164	1	164	1	164	1.90E-124	339	100
1l37A	2LZMA	99.39	164	1	164	1	164	4.02E-124	338	100
1l38A	2LZMA	99.39	164	1	164	1	164	2.10E-124	339	100
1l39A	2LZMA	98.171	164	1	164	1	164	7.46E-122	332	100
1l42A	2LZMA	99.39	164	1	164	1	164	2.08E-124	339	100
1l44A	2LZMA	99.39	164	1	164	1	164	3.37E-124	338	100
1l45A	2LZMA	99.39	164	1	164	1	164	2.08E-124	339	100
1l46A	2LZMA	99.39	164	1	164	1	164	2.08E-124	339	100
1l47A	2LZMA	99.39	164	1	164	1	164	3.37E-124	338	100
1l48A	2LZMA	99.39	164	1	164	1	164	1.99E-124	339	100
1l52A	2LZMA	99.39	164	1	164	1	164	9.86E-125	340	100
1l53A	2LZMA	99.39	164	1	164	1	164	3.09E-124	338	100
1l55A	2LZMA	98.148	162	1	162	1	162	1.52E-120	329	100
1l56A	2LZMA	99.39	164	1	164	1	164	4.53E-124	338	100
1l57A	2LZMA	99.383	162	1	162	1	162	7.41E-123	335	100
1l59A	2LZMA	98.148	162	1	162	1	162	2.30E-120	328	100
1l60A	2LZMA	99.39	164	1	164	1	164	3.05E-124	338	100
1l61A	2LZMA	98.148	162	1	162	1	162	1.57E-120	329	100
1l62A	2LZMA	98.148	162	1	162	1	162	3.35E-120	328	100

Query* ID	Subject * ID	% Identity	Alignment Length	Query start	Query end	Subject start	Subject end	E value	Bit score	% Coverage
1l65A	2LZMA	98.148	162	1	162	1	162	2.93E-120	328	100
1l66A	2LZMA	98.148	162	1	162	1	162	1.68E-120	329	100
1l67A	2LZMA	98.148	162	1	162	1	162	1.52E-120	329	100
1l68A	2LZMA	98.148	162	1	162	1	162	8.03E-121	330	100
1l69A	2LZMA	99.383	162	1	162	1	162	7.65E-123	335	100
1l76A	2LZMA	98.148	162	1	162	1	162	3.90E-120	328	100
1l77A	2LZMA	98.148	162	1	162	1	162	1.15E-120	329	100
1l85A	2LZMA	98.148	162	1	162	1	162	5.25E-120	328	100
1l86A	2LZMA	98.148	162	1	162	1	162	2.11E-120	328	100
1l87A	2LZMA	98.148	162	1	162	1	162	1.75E-120	329	100
1l88A	2LZMA	98.148	162	1	162	1	162	1.83E-120	329	100
1l90A	2LZMA	98.148	162	1	162	1	162	1.52E-120	329	100
1l91A	2LZMA	98.148	162	1	162	1	162	1.41E-120	329	100
1l92A	2LZMA	98.148	162	1	162	1	162	7.60E-121	330	100
1l93A	2LZMA	98.148	162	1	162	1	162	5.72E-121	330	100
1l94A	2LZMA	98.148	162	1	162	1	162	7.86E-121	330	100
1l95A	2LZMA	98.148	162	1	162	1	162	2.75E-120	328	100
1l96A	2LZMA	99.383	162	1	162	1	162	1.45E-122	334	100
1l98A	2LZMA	99.39	164	1	164	1	164	2.10E-124	339	100
1l99A	2LZMA	99.39	164	1	164	1	164	1.03E-123	337	100
1lavA	2RN2A	99.355	155	1	155	1	155	3.36E-119	325	100
1lawA	2RN2A	99.355	155	1	155	1	155	1.82E-119	325	100
1lhhA	1LZ1A	99.231	130	1	130	1	130	1.04E-96	266	100
1lhiA	1LZ1A	99.231	130	1	130	1	130	4.85E-96	264	100
1lhjA	1LZ1A	99.231	130	1	130	1	130	4.85E-96	264	100
1lhkA	1LZ1A	99.231	130	1	130	1	130	1.39E-96	266	100
1lhlA	1LZ1A	99.231	130	1	130	1	130	7.11E-97	266	100
1lsnA	4LYZA	98.45	129	1	129	1	129	7.48E-95	261	100
1lyeA	2LZMA	98.148	162	1	162	1	162	1.39E-120	329	100
1lyfA	2LZMA	98.148	162	1	162	1	162	8.58E-121	330	100
1lygA	2LZMA	98.148	162	1	162	1	162	2.30E-120	328	100
1lyhA	2LZMA	98.148	162	1	162	1	162	3.69E-120	328	100
1lyiA	2LZMA	98.148	162	1	162	1	162	3.35E-120	328	100
1lyjA	2LZMA	98.148	162	1	162	1	162	1.42E-120	329	100

Query* ID	Subject * ID	% Identity	Alignment Length	Query start	Query end	Subject start	Subject end	E value	Bit score	% Coverage
1mx2A	1IHBA	99.359	156	1	156	1	156	1.23E-113	311	100
1mx4A	1IHBA	99.359	156	1	156	1	156	9.02E-114	311	100
1mx6A	1IHBA	99.359	156	1	156	1	156	1.23E-113	311	100
1mykA	1ARRA	97.872	47	1	47	6	52	5.86E-31	94	100
1nagA	5PTIA	98.214	56	1	56	1	56	3.50E-39	115	100
1ouaA	1LZ1A	99.231	130	1	130	1	130	7.34E-97	266	100
1oubA	1LZ1A	99.231	130	1	130	1	130	6.65E-97	266	100
1oucA	1LZ1A	99.231	130	1	130	1	130	6.65E-97	266	100
1oudA	1LZ1A	99.231	130	1	130	1	130	6.65E-97	266	100
1oueA	1LZ1A	99.231	130	1	130	1	130	6.65E-97	266	100
1oufA	1LZ1A	100	129	1	129	1	129	6.65E-97	266	99.2
1ougA	1LZ1A	99.231	130	1	130	1	130	6.65E-97	266	100
1ouhA	1LZ1A	99.231	130	1	130	1	130	6.65E-97	266	100
1ouiA	1LZ1A	99.231	130	1	130	1	130	6.65E-97	266	100
1oujA	1LZ1A	99.231	130	1	130	1	130	6.65E-97	266	100
1p2lA	2LZMA	98.171	164	1	164	1	164	1.55E-122	334	100
1p2rA	2LZMA	98.171	164	1	164	1	164	1.39E-122	334	100
1p36A	2LZMA	98.171	164	1	164	1	164	1.39E-122	334	100
1p46A	2LZMA	98.171	164	1	164	1	164	4.55E-122	333	100
1p64A	2LZMA	98.171	164	1	164	1	164	3.90E-122	333	100
1p6yA	2LZMA	98.171	164	1	164	1	164	1.22E-121	332	100
1p7sA	2LZMA	98.171	164	1	164	1	164	1.55E-122	334	100
1qirA	1AMQA	99.747	396	1	396	1	396	0	819	100
1qisA	1AMQA	99.747	396	1	396	1	396	0	819	100
1qitA	1AMQA	99.747	396	1	396	1	396	0	819	100
1qs5A	2LZMA	98.148	162	1	162	1	162	2.66E-120	328	100
1qs9A	2LZMA	98.148	162	1	162	1	162	1.29E-120	329	100
1qsbA	2LZMA	98.148	162	1	162	1	162	2.96E-120	328	100
1qt6A	2LZMA	98.171	164	1	164	1	164	4.65E-122	333	100
1qt7A	2LZMA	98.171	164	1	164	1	164	8.24E-122	332	100
1qtbA	2LZMA	98.148	162	1	162	1	162	1.29E-120	329	100
1qtcA	2LZMA	98.148	162	1	162	1	162	3.82E-120	328	100
1qtdA	2LZMA	98.148	162	1	162	1	162	6.05E-120	327	100
1rbrA	2RN2A	99.355	155	1	155	1	155	3.22E-118	322	100

Query* ID	Subject * ID	% Identity	Alignment Length	Query start	Query end	Subject start	Subject end	E value	Bit score	% Coverage
1rbsA	2RN2A	99.355	155	1	155	1	155	2.27E-118	323	100
1rbtA	2RN2A	99.355	155	1	155	1	155	1.43E-118	323	100
1rbuA	2RN2A	99.355	155	1	155	1	155	6.93E-119	324	100
1rbvA	2RN2A	99.355	155	1	155	1	155	7.41E-119	324	100
1rdaA	2RN2A	99.355	155	1	155	1	155	5.88E-119	324	100
1rdbA	2RN2A	99.355	155	1	155	1	155	3.11E-119	325	100
1rdcA	2RN2A	99.355	155	1	155	1	155	5.88E-119	324	100
1rgcA	1RN1C	99.038	104	1	104	1	104	8.62E-76	211	100
1sycA	1EY0A	99.265	136	1	136	1	136	1.91E-100	276	100
1syeA	1EY0A	99.265	136	1	136	1	136	1.02E-100	276	100
1sygA	1EY0A	99.265	136	1	136	1	136	1.02E-100	276	100
1tlaA	2LZMA	98.148	162	1	162	1	162	3.99E-120	328	100
1varA	1N0JA	99.495	198	1	198	1	198	1.35E-151	410	100
1vqgA	1VQBA	98.837	86	1	86	1	86	9.77E-63	177	100
1vqhA	1VQBA	98.837	86	1	86	1	86	1.04E-62	177	100
1vqiA	1VQBA	98.837	86	1	86	1	86	5.70E-63	177	100
1vqjA	1VQBA	98.837	86	1	86	1	86	5.77E-63	177	100
1wqmA	1LZ1A	99.231	130	1	130	1	130	4.15E-97	267	100
1wqnA	1LZ1A	99.231	130	1	130	1	130	4.15E-97	267	100
1wqoA	1LZ1A	99.231	130	1	130	1	130	4.15E-97	267	100
1wqpA	1LZ1A	99.231	130	1	130	1	130	4.15E-97	267	100
1wqqA	1LZ1A	99.231	130	1	130	1	130	4.15E-97	267	100
1wqrA	1LZ1A	99.231	130	1	130	1	130	4.15E-97	267	100
1yamA	1LZ1A	99.231	130	1	130	1	130	2.62E-97	268	100
1yanA	1LZ1A	99.231	130	1	130	1	130	2.62E-97	268	100
1yaoA	1LZ1A	99.231	130	1	130	1	130	2.62E-97	268	100
1yapA	1LZ1A	99.231	130	1	130	1	130	2.62E-97	268	100
1yaqA	1LZ1A	99.231	130	1	130	1	130	2.62E-97	268	100
1yhbA	1VQBA	98.837	86	1	86	1	86	8.83E-63	177	98.9
206lA	2LZMA	98.148	162	1	162	1	162	7.20E-121	330	100
217lA	2LZMA	98.148	162	1	162	1	162	1.71E-120	329	100
221lA	2LZMA	98.148	162	1	162	1	162	7.20E-121	330	100
224lA	2LZMA	98.148	162	1	162	1	162	7.20E-121	330	100
230lA	2LZMA	98.171	164	1	164	1	164	3.62E-122	333	100

Query* ID	Subject * ID	% Identity	Alignment Length	Query start	Query end	Subject start	Subject end	E value	Bit score	% Coverage
231lA	2LZMA	98.148	162	1	162	1	162	3.73E-120	328	100
232lA	2LZMA	98.148	162	1	162	1	162	3.73E-120	328	100
233lA	2LZMA	98.148	162	1	162	1	162	1.15E-120	329	100
234lA	2LZMA	98.148	162	1	162	1	162	1.15E-120	329	100
235lA	2LZMA	98.148	162	1	162	1	162	1.32E-120	329	100
236lA	2LZMA	98.148	162	1	162	1	162	1.32E-120	329	100
237lA	2LZMA	98.171	164	1	164	1	164	4.04E-122	333	100
238lA	2LZMA	98.148	162	1	162	1	162	1.32E-120	329	100
239lA	2LZMA	98.148	162	1	162	1	162	1.41E-120	329	100
240lA	2LZMA	98.148	162	1	162	1	162	1.41E-120	329	100
241lA	2LZMA	98.148	162	1	162	1	162	1.41E-120	329	100
242lA	2LZMA	98.171	164	1	164	1	164	5.08E-122	333	100
243lA	2LZMA	98.148	162	1	162	1	162	1.41E-120	329	100
244lA	2LZMA	98.148	162	1	162	1	162	1.41E-120	329	100
245lA	2LZMA	98.171	164	1	164	1	164	8.61E-122	332	100
246lA	2LZMA	98.171	164	1	164	1	164	1.52E-121	332	100
247lA	2LZMA	98.171	164	1	164	1	164	5.08E-122	333	100
253lA	2LZMA	98.171	164	1	164	1	164	1.14E-121	332	100
254lA	2LZMA	98.171	164	1	164	1	164	5.73E-122	333	100
255lA	2LZMA	98.171	164	1	164	1	164	4.97E-122	333	100
2exzA	1EY0A	99.259	135	1	135	2	136	1.38E-100	276	100
2ey1A	1EY0A	99.265	136	1	136	1	136	4.53E-101	278	100
2ey2A	1EY0A	99.265	136	1	136	1	136	4.20E-101	278	100
2ey5A	1EY0A	99.259	135	1	135	2	136	9.80E-101	276	100
2ey6A	1EY0A	99.259	135	1	135	2	136	1.87E-100	276	100
2eyfA	1EY0A	99.259	135	1	135	2	136	1.87E-100	276	100
2eyhA	1EY0A	99.259	135	1	135	2	136	9.80E-101	276	100
2eyjA	1EY0A	99.265	136	1	136	1	136	4.53E-101	278	100
2eylA	1EY0A	99.265	136	1	136	1	136	1.82E-101	278	100
2eymA	1EY0A	99.259	135	1	135	2	136	1.38E-100	276	100
2eyoA	1EY0A	99.259	135	1	135	2	136	9.80E-101	276	100
2eypA	1EY0A	99.265	136	1	136	1	136	4.53E-101	278	100
2f0dA	1EY0A	99.265	136	1	136	1	136	1.18E-101	279	100
2f0eA	1EY0A	99.259	135	1	135	2	136	1.13E-100	276	100

Query* ID	Subject * ID	% Identity	Alignment Length	Query start	Query end	Subject start	Subject end	E value	Bit score	% Coverage
2f0fA	1EY0A	99.265	136	1	136	1	136	1.58E-101	279	100
2f0gA	1EY0A	99.259	135	1	135	2	136	5.54E-101	277	100
2f0hA	1EY0A	99.259	135	1	135	2	136	1.13E-100	276	100
2f0iA	1EY0A	99.265	136	1	136	1	136	2.37E-101	278	100
2f0jA	1EY0A	99.259	135	1	135	2	136	6.53E-101	277	100
2heaA	1LZ1A	99.231	130	1	130	1	130	1.01E-96	266	100
2hebA	1LZ1A	99.231	130	1	130	1	130	1.01E-96	266	100
2hecA	1LZ1A	99.231	130	1	130	1	130	1.01E-96	266	100
2hedA	1LZ1A	99.231	130	1	130	1	130	1.01E-96	266	100
2heeA	1LZ1A	99.231	130	1	130	1	130	2.54E-96	265	100
2hefA	1LZ1A	99.231	130	1	130	1	130	1.01E-96	266	100
2l78A	2LZMA	98.148	162	1	162	1	162	4.74E-121	330	100
2snmA	1EY0A	99.259	135	1	135	2	136	5.31E-100	275	100
3aa2A	2RN2A	99.346	153	1	153	3	155	4.42E-117	319	100
3aa3A	2RN2A	99.355	155	1	155	1	155	9.75E-119	324	100
3aa4A	2RN2A	99.342	152	1	152	2	153	1.48E-116	318	100
3aa5X	2RN2A	99.346	153	1	153	2	154	4.67E-117	319	100
3c7wA	2LZMA	99.39	164	1	164	1	164	1.26E-124	339	100
3c7yA	2LZMA	99.39	164	1	164	1	164	5.58E-124	338	100
3c80A	2LZMA	99.383	162	1	162	1	162	1.61E-122	334	100
3c81A	2LZMA	99.39	164	1	164	1	164	3.76E-124	338	100
3c83A	2LZMA	99.39	164	1	164	1	164	6.88E-124	337	100
3c8qA	2LZMA	99.39	164	1	164	1	164	6.80E-124	337	100
3c8rA	2LZMA	99.39	164	1	164	1	164	9.77E-124	337	100
3c8sA	2LZMA	99.39	164	1	164	1	164	3.37E-124	338	100
3cdoA	2LZMA	99.383	162	1	162	1	162	1.53E-122	334	100
3cdqA	2LZMA	99.39	164	1	164	1	164	3.19E-124	338	100
3cdrA	2LZMA	99.39	164	1	164	1	164	1.97E-124	339	100
3cdtA	2LZMA	99.39	164	1	164	1	164	4.15E-124	338	100
3cdvA	2LZMA	99.39	164	1	164	1	164	4.74E-124	338	100
3f8vA	2LZMA	99.39	164	1	164	1	164	2.92E-124	338	100
3fi5A	2LZMA	99.39	164	1	164	1	164	1.52E-123	337	100
3nwvA					No significant hits					
4d6bA	1HMSA	62.791	129	4	132	2	130	6.16E-60	173	97

Query* ID	Subject * ID	% Identity	Alignment Length	Query start	Query end	Subject start	Subject end	E value	Bit score	% Coverage
5eaaA	1AMQA	99.747	396	1	396	1	396	0	820	100
5n4mA	1HMSA	62.791	129	4	132	2	130	1.58E-60	175	97
5n4pA	1HMSA	63.566	129	4	132	2	130	4.16E-61	176	97
5n4qA	1HMSA	62.791	129	4	132	2	130	6.04E-61	176	97
5o10A					No significant hits					
6ew5A	1HMSA	62.791	129	4	132	2	130	1.85E-60	174	97
8ptiA	5PTIA	98.276	58	1	58	1	58	3.59E-40	117	100

*Query ID: PDB ID + Chain ID for proteins in the Ssym+ reverse dataset

*Subject ID: PDB ID + Chain ID for best hit in the S2298 dataset
